# Bromophosphatation as a Mode of Chiral Phosphoric Acid Catalyst Deactivation as Elucidated by Kinetic Profiling

**DOI:** 10.1021/acs.joc.5c00431

**Published:** 2025-05-20

**Authors:** Ben M. J. Lancaster, Andrew J. P. White, Christopher J. Tighe, D. Christopher Braddock

**Affiliations:** † Department of Chemistry, Imperial College London, Molecular Sciences Research Hub, White City Campus, 82 Wood Lane, London W12 0BZ, U.K.; ‡ Department of Chemical Engineering, Imperial College London, South Kensington Campus, Imperial College Road, London SW7 2AZ, U.K.

## Abstract

A BINOL-derived chiral phosphoric acid (*R*)-**1** was shown by kinetic profiling to be deactivated during the catalytic bromoesterification of cyclohexene. The products of the deactivation were identified as diastereoisomeric phosphates (*R*,1*R*,2*R*)-**3a** and (*R*,1*S*,2*S*)-**3b** and are formed via an alkene bromophosphatation process where the phosphate of **1** behaves as a competitive nucleophile, as confirmed by authentic preparations of **3a** and **3b** from a stoichiometric bromophosphatation reaction. HPLC separation of the diastereoisomers gave pure **3a** whose absolute and relative configurations were proven by single-crystal X-ray diffraction. The ^31^P­{^1^H} NMR spectrum of phosphate **3a** displayed four resonances despite **3a** having just one phosphorus atom, and combined VT-NMR and DFT analysis revealed this to be a consequence of rotational isomerism about the 9-phenanthrene (Ar) bearing C3,3′–Ar bonds. Moreover, kinetic studies using variable time normalization analysis (VTNA) of the catalytic cyclohexene bromoesterification showed first order kinetics in all reactants. The amount of phosphates **3a** and **3b** formed under catalytic bromoesterification conditions was quantified, enabling tracking of the temporal catalyst **1** concentration and hence elucidation of first order kinetics in catalyst **1**. A catalytic cycle consistent with these observations is proposed.

## Graphical abstract

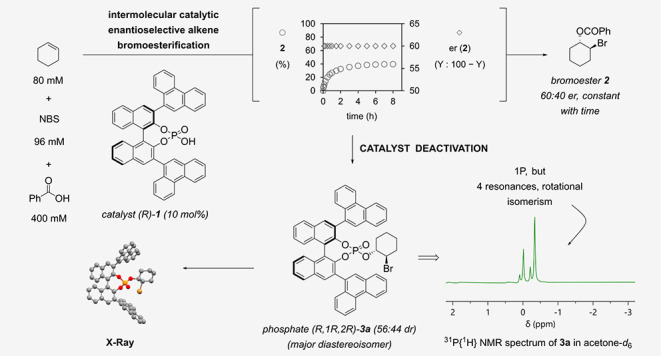

## Introduction

The intermolecular alkene bromoesterification reaction is a direct and versatile approach for the formation of vicinal, stereogenic C–Br and C–O bonds.^1^ Since 2007, several examples have been developed using achiral catalysts,^2^ but successful enantioselective variants utilizing chiral catalysts remain underdeveloped.^3^ In 2012, Tang et al.^3a^ reported the first intermolecular, enantioselective alkene bromoesterification reaction utilizing 10 mol % of BINOL-derived chiral phosphoric acid catalyst (*R*)-**1** ^4^ (Scheme 1). According to Tang et al., various aromatic bromoesters **2**-Ar were prepared in low to moderate er, although in only 10–20% isolated yield. Tang et al. speculated that the low yield might be attributable to a temporal decline in catalyst loading by nucleophilic addition of the phosphate of **1** to a putative bromonium ion. However, no structure was provided for the resultant bromoalkylated phosphate nor were any characterizing data provided.

**Scheme 1:**
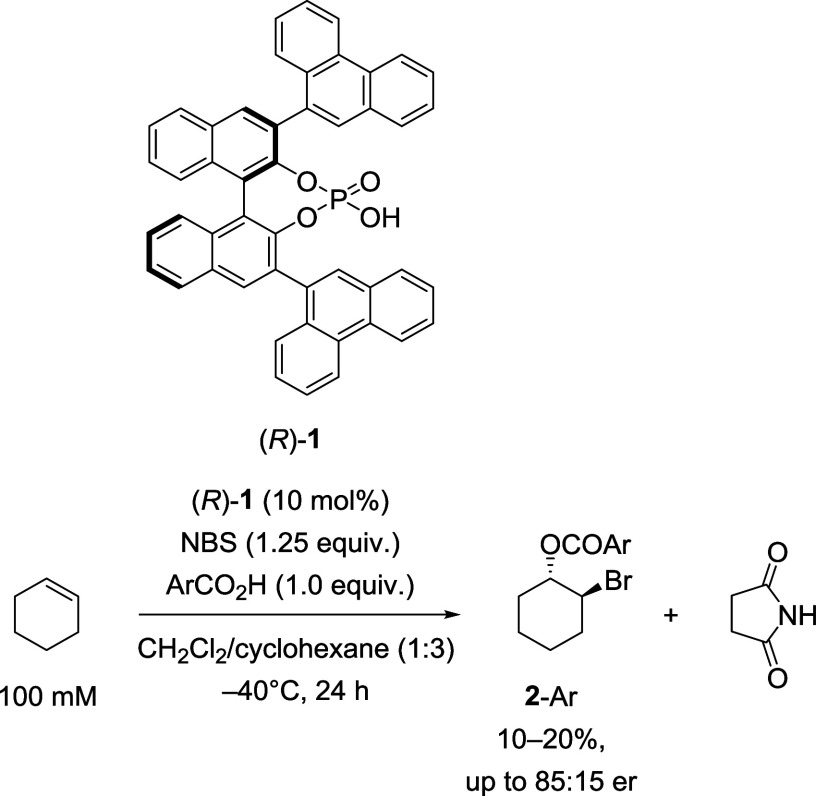
Bromoesterification of Cyclohexene to Form Bromoesters **2**-Ar as Reported by Tang et al.^3a^

In 2023, we reported that an intermolecular alkene bromoesterification reaction catalyzed by (DHQD)_2_PHAL and utilizing PhCONHBr as the stochiometric electrophilic bromination reagent (as previously reported by Shi et al.)^3c^ was inhibited by the benzamide byproduct.^5^ Therefore, we considered if inhibition by the succinimide byproduct in Tang et al.’s reaction might be a suitable alternative explanation for the low reaction yields. Herein, we use kinetic profiling methods and time-adjusted analysis to demonstrate that this is not the case and that the phosphoric acid **1** catalyzed bromoesterification of cyclohexene is indeed impeded by deactivation of the catalyst. Moreover, we disclose the preparation of the authentic catalyst deactivation products, to wit, diastereoisomeric phosphates **3a** and **3b** (Figure 1), by the bromoalkylation of phosphoric acid **1**. X-ray diffraction studies of diastereoisomer (*R*,1*R*,2*R*)-**3a** allowed its absolute and relative configurations to be established. Furthermore, the formation of phosphates **3a** and **3b** under standard catalytic bromoesterification conditions was quantified.

**Figure 1:**
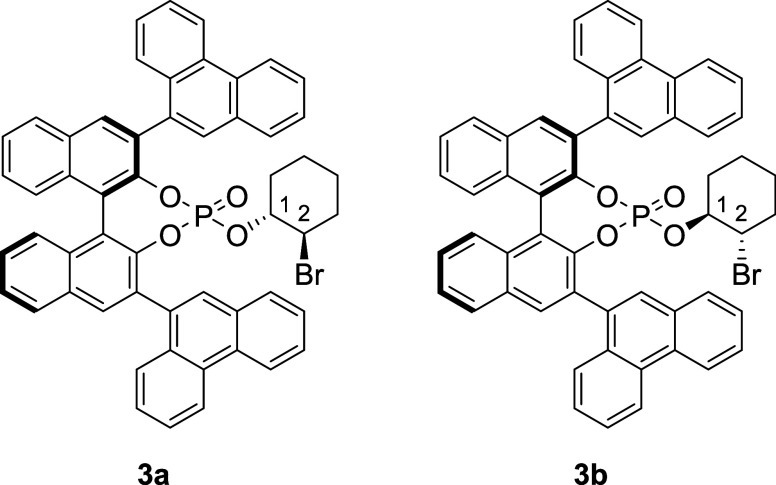
Diastereoisomeric phosphates **3a** and **3b**, which arise from deactivation of Catalyst **1**.

## Results and Discussion

Tang et al.’s phosphoric acid (*R*)-**1** catalyzed bromobenzoylation of cyclohexene with benzoic acid was selected as a representative reaction for kinetic investigation. According to Tang et al., bromoester **2** was isolated in 15% yield and 77.5:22.5 er from a mixture of 1.25 equiv of NBS, 1.0 equiv of benzoic acid and cyclohexene, and 10 mol % of catalyst **1**, in CH_2_Cl_2_ and cyclohexane (*v*/*v* = 1:3, 0.1 M), reacted at −40 °C for 24 h (Scheme 1). However, these conditions did not yield a homogeneous reaction mixture when attempted in our laboratory. Consequently, the first stage of our investigation was to alter the protocol to make the reaction mixture homogeneous and thus amenable to kinetic analysis: the NBS and benzoic acid were adjusted to 1.2 equiv, CH_2_Cl_2_ was employed as the solvent, the temperature was raised to 0 °C, and the reaction mixture was diluted to 80% of its original concentration. Second, a method was developed to assess the temporal formation of bromoester **2**; viz., aliquots were withdrawn from the reaction, immediately quenched, and analyzed by HPLC with 4,4’-dimethylbenzophenone added as an internal standard.^6^ Having developed a protocol for kinetic analysis, an experiment was performed using the specified conditions (Scheme 2). HPLC analysis revealed that after 8 h under these conditions, bromoester **2** was formed in 17% conversion and 60:40 er. These results demonstrate a catalytic performance similar to that of Tang et al., albeit with a decrease in er attributable to the increase in temperature, and the bromoesterification is expected to occur in the same mechanistic manifold in both cases.

**Scheme 2:**
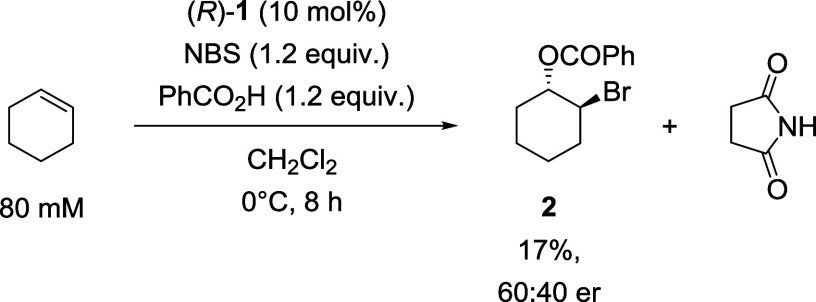
Bromoesterification of Cyclohexene to Form Bromoester **2** Using Modified Conditions

The final objective before commencing our kinetic study was to raise the conversion to bromoester **2**.^7^ Therefore, the influence of benzoic acid on the conversion was investigated by administering an excess of 2.0, 3.5, 5.0, and 10.0 equiv (at 10.0 equiv, the benzoic acid did not fully dissolve). The initial rate of reaction increased in each case as a result, and higher conversions to bromoester **2** were attained over 8 h (Figure 2). Moreover, the er of bromoester **2** in all these reactions remained at 60:40 and did not change with time.^8^ The experiment utilizing a 5.0 equiv excess of benzoic acid was chosen as the representative standard conditions for subsequent experiments.

**Figure 2:**
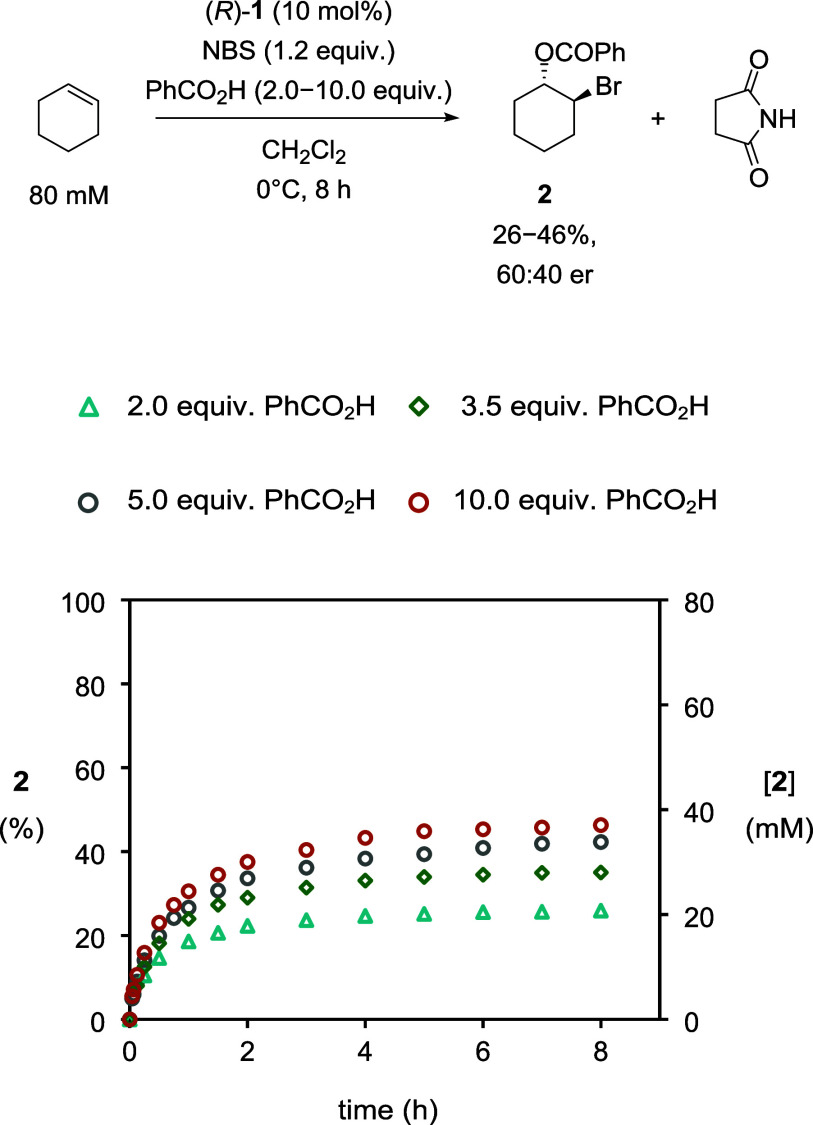
Plot of conversion to **2** (%) and [**2**] vs time in experiments of varying benzoic acid concentrations, as monitored by HPLC methods. [cyclohexene]_0_ = 80 mM, [NBS]_0_ = 96 mM, and [**1**]_0_ = 8 mM. (i) △ (blue), with [PhCO_2_H]_0_ = 160 mM. (ii) ◇ (green), with [PhCO_2_H]_0_ = 280 mM. (iii) ○ (gray), with [PhCO_2_H]_0_ = 400 mM. (iv) ○ (orange) (heterogeneous), with [PhCO_2_H]_0_ ∼650 mM.

To determine if the Brønsted acidity of catalyst **1** was an important attribute of catalysis, an experiment was conducted in which the catalyst was replaced by 10 mol % of the sodium phosphate salt of **1**.^9^ HPLC analysis after 8 h indicated that (racemic) bromoester *rac*-**2** formed in just 2% conversion. The same conversion was also obtained on the exclusion of the catalyst from the bromoesterification reaction; evidently, **1** must be in its acidic form for catalysis to proceed. Next, we explored the effect of varying the loading of catalyst **1** (originally 10 mol %) (Figure 3a): when loading was reduced to 6 mol %, the initial reaction rate and conversion to bromoester **2** fell; in contrast, at a greater loading of 20 mol %, the initial reaction rate improved, and an increased conversion to **2** was realized over 8 h.^10^

**Figure 3:**
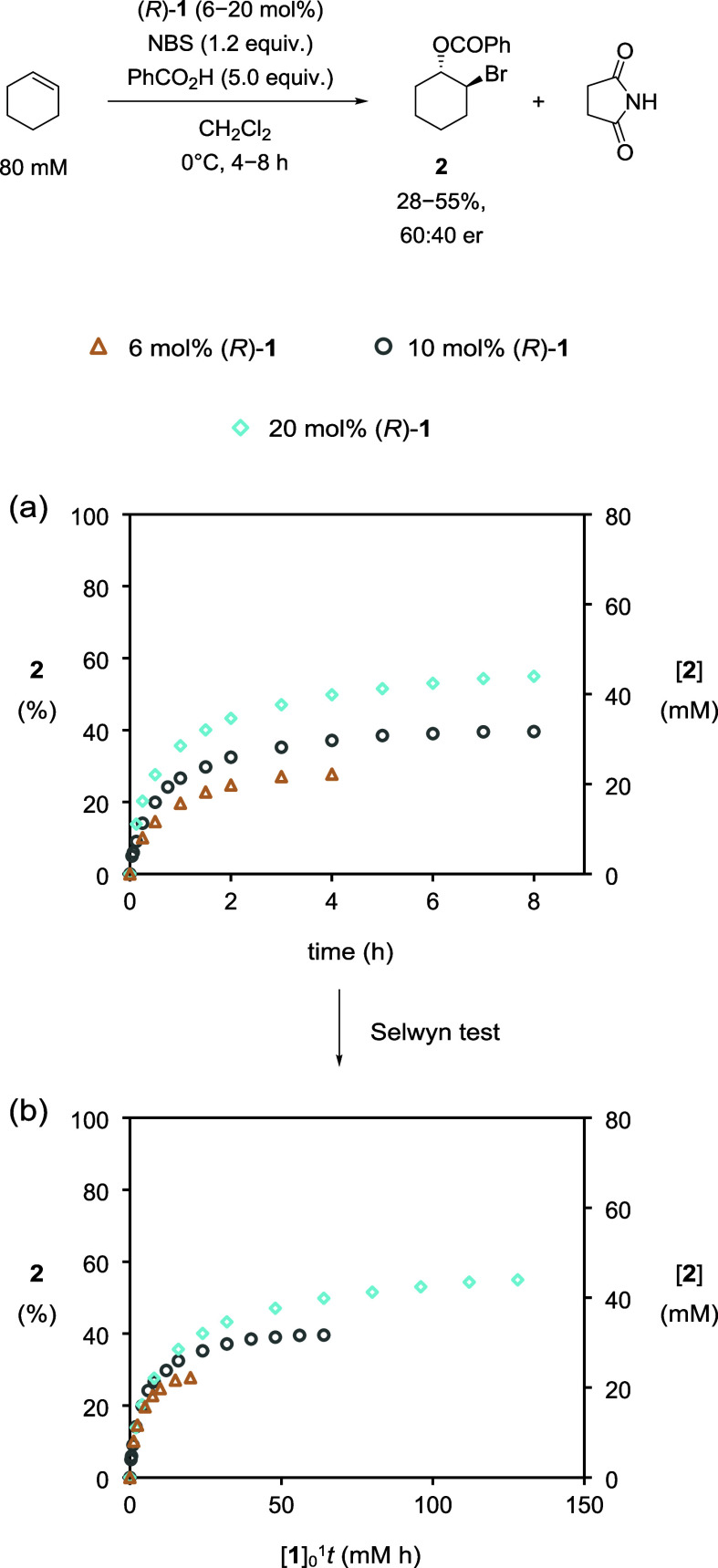
Plots of (a) conversion to **2** (%) and [**2**] vs time and (b) conversion to **2** (%) and [**2**] vs normalized time in experiments with varying catalyst loadings as monitored by HPLC methods. [cyclohexene]_0_ = 80 mM, [PhCO_2_H]_0_ = 400 mM, and [NBS]_0_ = 96 mM. (i) ○ (gray), with [**1**]_0_ = 8 mM. (ii) ◇ (blue), with [**1**]_0_ = 16 mM. (iii) △ (brown), with [**1**]_0_ = 5 mM.

It is apparent from an inspection of the profiles that over half of the conversion to the final bromoester **2** concentration takes place within the first hour, after which the reaction rate slows considerably. Therefore, we were keen to investigate the stability of catalyst **1** by applying the Selwyn test to Figure 3a.^11^ Selwyn’s test involves recasting the profiles of varying catalyst loading with an abscissa of time multiplied by the initial concentration of **1**. If catalyst **1** is stable, then its concentration will be independent of time, and the normalized profiles will overlay. However, once normalization had been applied, the profiles did not overlay across the full reaction time scale (Figure 3b). This suggests that catalyst **1** is not stable during the reaction and is falling in concentration as the reaction proceeds.^12^

The time-adjusted analysis protocol set forth by Blackmond et al.^13^ was utilized to reinforce the finding of catalyst deactivation and to rule out byproduct inhibition. The experiment at 10 mol % catalyst loading was chosen as a representative standard, and a same excess experiment was conducted with initial concentrations of NBS, cyclohexene, and benzoic acid reduced by 20 mM; this simulates 25% completion of the standard experiment, albeit crucially without the presence of products. If neither catalyst deactivation nor product inhibition is occurring, then the same excess plot will overlay with its standard partner once two corrections are applied: (i) adjustment of the same excess time axis by 0.8 h, which corresponds to the time elapsed in the standard experiment for 25% conversion, and (ii) increase of the ordinate by 25% to account for bromoester **2** formed during this time.^14^ However, the two profiles did not overlay (Figure 4a), with instead the experiment under the same excess conditions proceeding at faster rate than the standard experiment at the same concentration.

**Figure 4:**
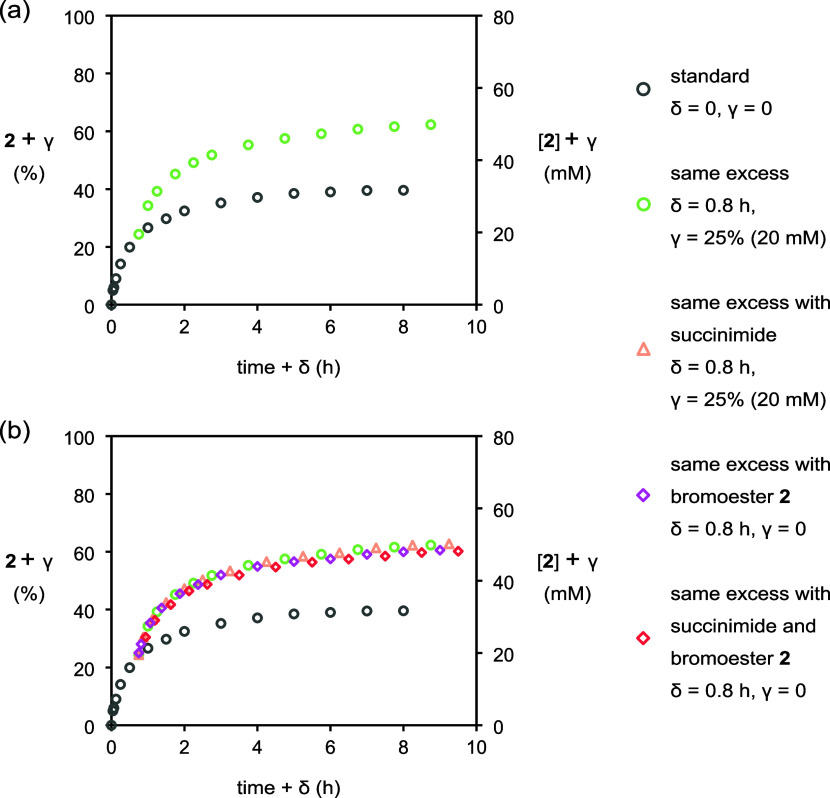
Plot of conversion to **2** (%) and [**2**] vs time in same excess experiments as monitored by HPLC methods. (i) ○ (gray), [cyclohexene]_0_ = 80 mM, [PhCO_2_H]_0_ = 400 mM, [NBS]_0_ = 96 mM, and [**1**]_0_ = 8 mM. (ii) ○ (green), [cyclohexene]_0_ = 60 mM, [PhCO_2_H]_0_ = 380 mM, [NBS]_0_ = 76 mM, and [**1**]_0_ = 8 mM. (iii) △ (orange), as for ○ (green) with [succinimide]_0_ = 20 mM. (iv) ◇ (purple), as for ○ (green) with [*rac*-**2**]_0_ = 20 mM. (v) ◇ (red), as for ○ (green) with [succinimide]_0_ = 20 mM, and [*rac*-**2**]_0_ = 20 mM.

Next, we conducted three iterations of the same excess experiment with the products added: (a) with byproduct succinimide added, (b) with bromoester **2** added, and (c) with both succinimide and **2** added. The profiles (Figure 4b) measured for all three of these experiments did not overlay with the standard experiment but did overlay with the same excess experiment lacking added products. This indicates that neither succinimide nor bromoester **2** depletes the reaction rate and confirms that deactivation of catalyst **1** is responsible for the slowing reaction rate.

Now that the deactivation of the catalyst had been demonstrated—and byproduct inhibition had been excluded—the focus shifted to identifying the product(s) formed by this process. It seemed plausible that the phosphate of **1** could compete with benzoic acid during nucleophilic addition, forming a mixture of (*R*,1*R*,2*R*)-**3a** and (*R*,1*S*,2*S*)-**3b** bromoalkylated phosphate diastereoisomers. Hence, a reaction was devised to form phosphates **3a** and **3b** by reacting a stoichiometric quantity of phosphoric acid **1** with NBS and cyclohexene, in the absence of benzoic acid but under conditions otherwise comparable to our kinetic analyses (Scheme 3). Gratifyingly, from this bromophosphatation process, phosphates **3a** and **3b** were isolated as a mixture in a combined 48% yield.

**Scheme 3:**
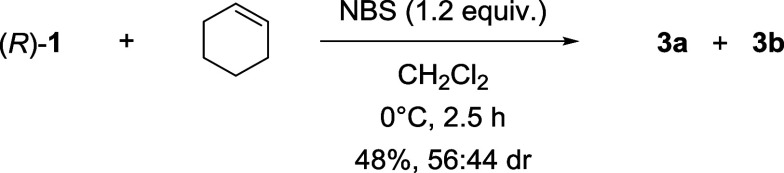
Bromophosphatation of Cyclohexene to Form Bromoalkylated Phosphates **3a** and **3b**

The HPLC chromatogram of the **3a** and **3b** phosphate mixture, measured with an achiral stationary phase, showed two broad peaks, which integrated to a 56:44 ratio. However, the ^31^P­{^1^H} NMR spectrum of the mixture in acetone-*d*_6_ exhibited 8 resonances (Figure 5). This was unexpected, as each of the phosphates **3a** and **3b** has just one phosphorus atom. Hence, authentic ^31^P­{^1^H} NMR spectra of the purified diastereoisomers were sought for further investigation. The diastereoisomers were inseparable by flash chromatography, although preparative HPLC proved suitable for separation, allowing isolation of pure phosphate diastereoisomer **3a** and 91:9 dr diastereoisomer **3b**. X-ray diffraction measurements of **3a**, following recrystallization by slow diffusion of cyclohexane into a saturated solution in acetone,^15^ confirmed its absolute and relative configurations (Figure 6). The ^31^P­{^1^H} NMR spectra of **3a** and **3b** (91:9 dr) (Figure 5) had four (major) resonances each; resonances corresponding to the former diastereoisomer were ∼1 ppm more deshielded than those of the latter. Moreover, when each pair of four resonances was integrated in the ^31^P­{^1^H} NMR spectrum of the mixture, a 56:44 ratio was obtained (Figure 5), directly comparable to the ratio measured by HPLC and therefore confirming this as the dr.

**Figure 5:**
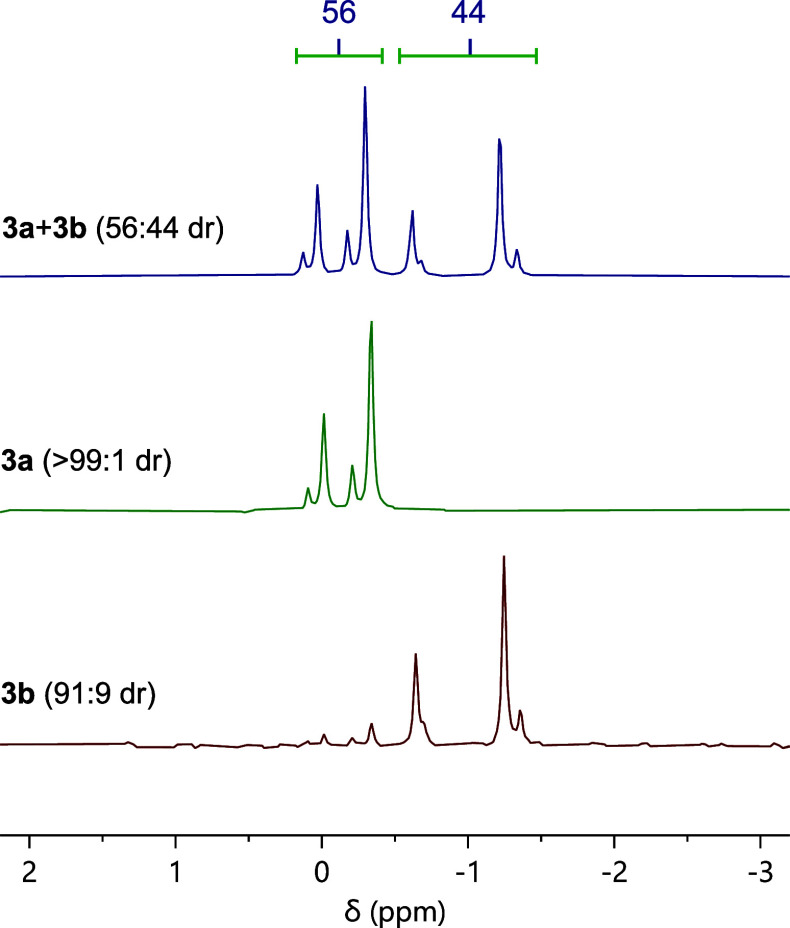
^31^P­{^1^H} NMR spectra of bromoalkylated phosphates **3a** and **3b** in acetone-*d*_6_.

**Figure 6:**
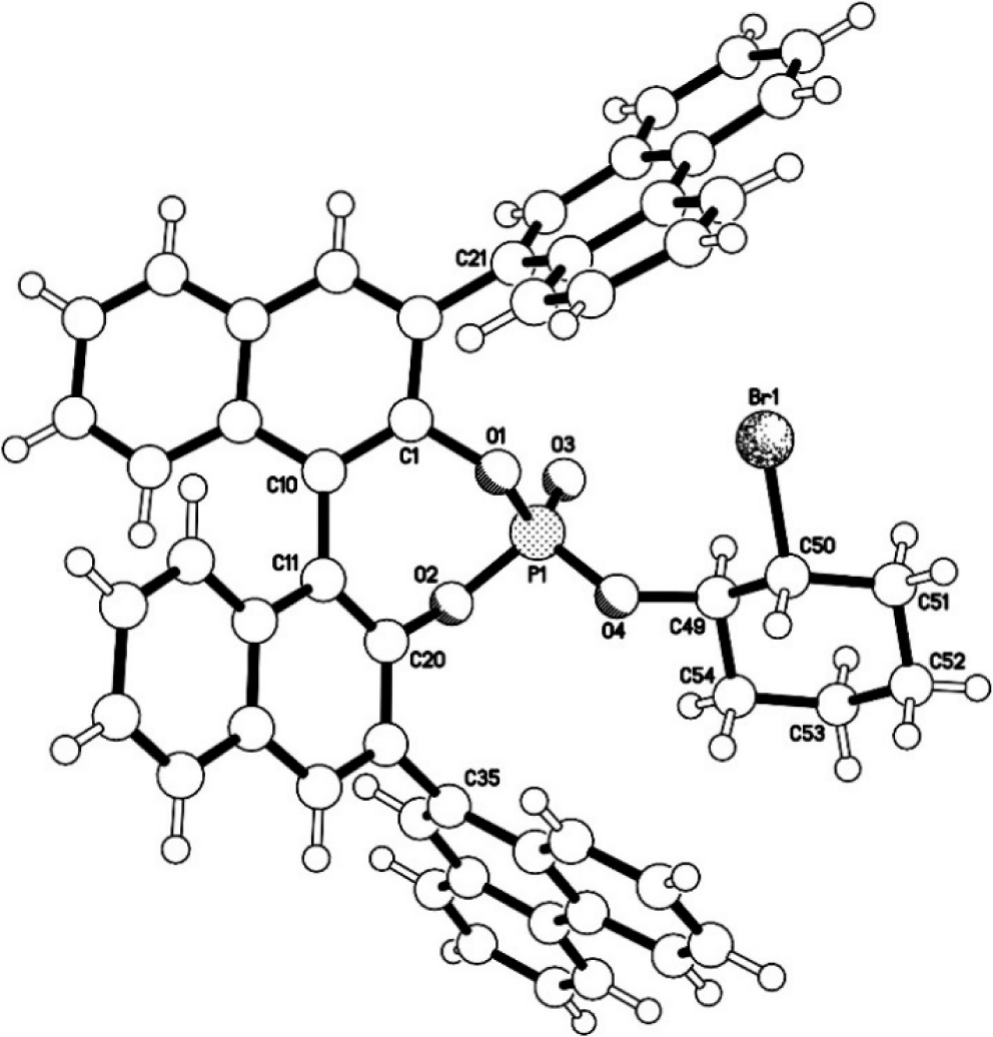
X-ray crystal structure of bromoalkylated phosphate diastereoisomer **3a**.

The observation of four resonances in the ^31^P­{^1^H} NMR spectra of each **3a** and **3b** phosphate diastereoisomer could be attributable to hindered rotation about their unsymmetrical 9-phenanthrene groups, giving rise to rotational isomers (Figure 7a). VT ^31^P­{^1^H} NMR studies were conducted to investigate this further. Accordingly, 56:44 dr phosphates **3a** and **3b** in DMSO-*d*_6_ were heated to 100 °C, which caused the seven observable resonances in this solvent at room temperature to collapse into four broad resonances; on cooling, these resonances reverted back to the original seven (for all spectra, see the Supporting Information). This experiment demonstrates at least partial conformational exchange on the NMR time scale at this temperature, although operational limitations prevented us from reaching full coalescence at higher temperatures. Therefore, we also opted to conduct DFT studies: in Gaussian 16W, three further orientations of the 9-phenanthrene groups in **3a** were modeled by uploading the X-ray diffraction structure, and adjusting one, or both, torsion angles ω_a_ and ω_b_ by 180 degrees. DFT with the B3LYP/IEFPCM/def2-SVP level of theory was used to optimize the structure of each rotamer, and the corresponding potential energies and Boltzmann populations were calculated at 295 K.^16^ Gratifyingly, a comparison of the integrals of the four peaks in the ^31^P­{^1^H} NMR spectrum of phosphate **3a** (Figure 7b) with the DFT calculated Boltzmann populations (Figure 7c) shows an excellent match with, at most, a difference of 3% (ii). Therefore, we attribute the four ^31^P­{^1^H} NMR resonances for phosphates **3a** and **3b** to 180-degree rotations about ω_a_ and ω_b_, which occur slowly on the NMR time scale and give rise to 2^2^ unique phosphorus environments for each diastereoisomer. This is the first DFT study on rotational isomerism in a 3,3′-diaryl-*O*,*O*’-disubstituted BINOL derivative.^17^

**Figure 7:**
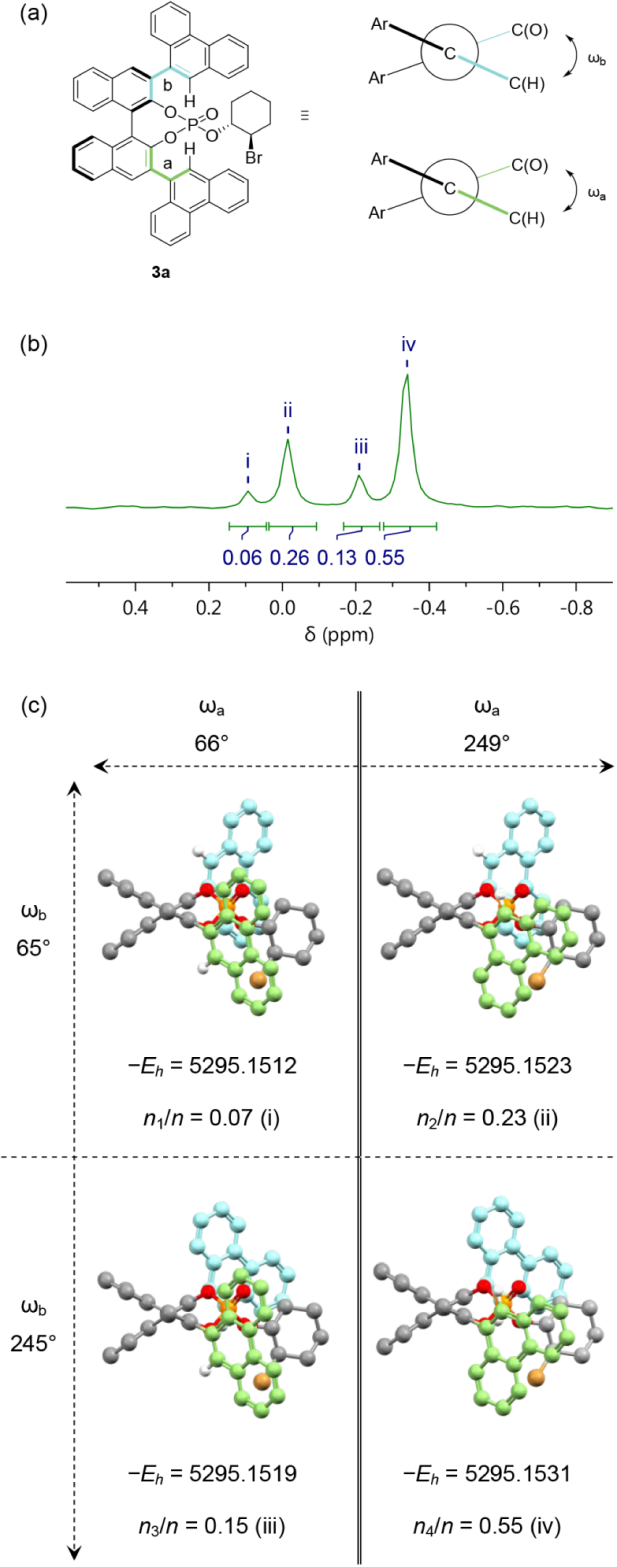
(a) Newman projection of phosphate **3a** with torsion angles ω_a_ and ω_b_ defined. (b) ^31^P­{^1^H} NMR spectrum of phosphate **3a** in acetone-*d*_6_ with relative integrals summing to 1P. (c) DFT calculated structures for rotational isomers of phosphate **3a** using the B3LYP/IEFPCM/def2-SVP (acetone) density functional; potential energies (*E*_*h*_) are provided in Hartree, with Boltzmann populations (*n*_*i*_/*n*) expressed summing to 1. ω_a_ = 249°, ω_b_ = 65° represents the experimentally determined X-ray diffraction structure.

Having isolated and characterized phosphates **3a** and **3b**, they were investigated as possible side products arising from the deactivation of catalyst **1** during the bromoesterification of cyclohexene. Thus, HPLC was utilized to analyze the temporal formation of phosphates **3a** and **3b** simultaneously with bromoester **2** under the standard conditions (Figure 8). Indeed, catalyst **1** underwent bromoalkylation to yield **3a** and **3b**, consuming more than half of catalyst **1** after 1 h and consequently severely retarding the desired enantioselective alkene bromobenzoylation.^18^ This result confirms that the low yield of the catalytic bromoesterification of cyclohexene can be attributed to deactivation of phosphoric acid **1** to phosphates **3a** and **3b**. The dr of **3a** and **3b** was 56:44, respectively, and did not vary with time; this is the same ratio as obtained from the bromophosphatation reaction presented earlier in the absence of benzoic acid.

**Figure 8:**
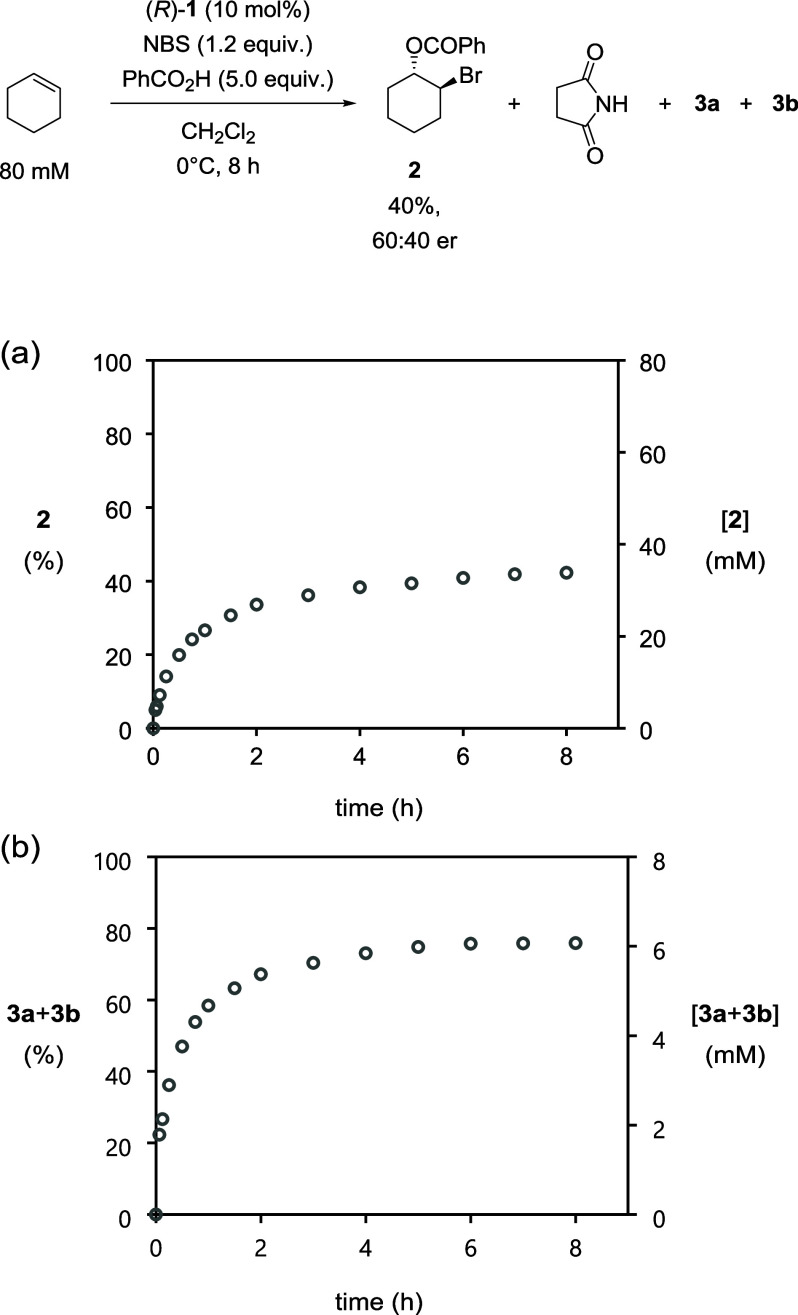
Plots of (a) conversion to **2** (%) and [**2**] vs time and (b) conversion to **3a**+**3b** (%) and [**3a**+**3b**] vs time as monitored by HPLC methods. [cyclohexene]_0_ = 80 mM, [PhCO_2_H]_0_ = 400 mM, [NBS]_0_ = 96 mM, and [**1**]_0_ = 8 mM.

The ability to monitor the deactivation of catalyst **1** enabled a complete kinetic study to determine the orders in the reaction components for the catalytic bromoesterification reaction. Thus, a series of different excess experiments were conducted where the stoichiometry of each reactant was varied (for conditions and all profiles, see the Supporting Information). Burés’ variable time normalization analysis (VTNA) procedure was applied to overlay the profiles of each experiment with the standard partner and hence determine reactant orders.^19^ NBS, cyclohexene, and benzoic acid^20^ each displayed first order kinetics using this method. The order in catalyst **1** was particularly of interest to validate the existing proposed catalytic cycle,^3a^ and so the VTNA method was also applied to our earlier experiments where catalyst **1** loading was varied (Figure 3). To our delight, all three profiles overlaid across the full range when the time scale was normalized with the first power of the temporal catalyst **1** concentration, calculated by subtracting the concentration of phosphates **3a** and **3b** from the initial concentration of **1** (Figure 9). This indicates that catalyst **1** is also first order and supplants the earlier Selwyn analysis. Overall, the VTNA results indicate that NBS, cyclohexene, benzoic acid, and catalyst **1** are involved up to and including the rate-determining step.^21^

**Figure 9:**
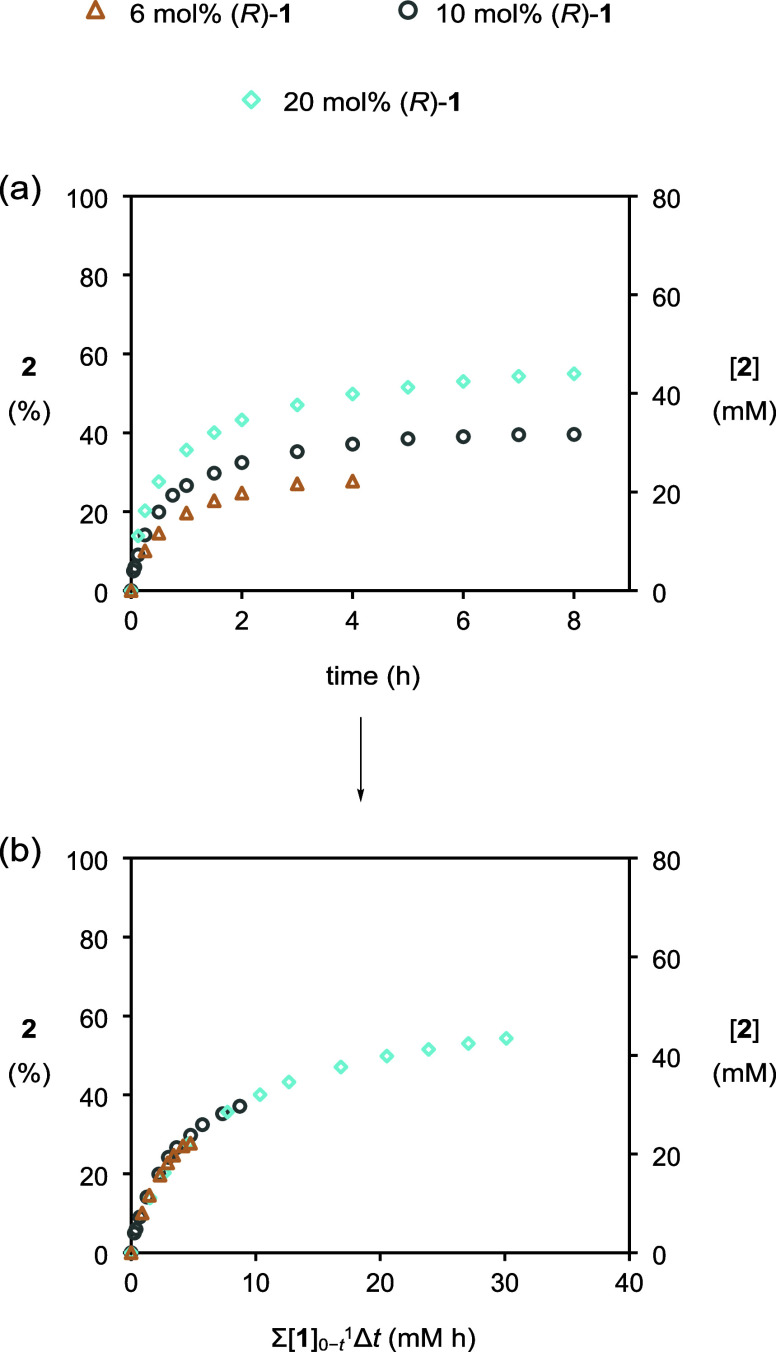
Plots of (a) conversion to **2** (%) and [**2**] vs time and (b) conversion to **2** (%) and [**2**] vs normalized time in experiments with varying catalyst loadings as monitored by HPLC methods. [cyclohexene]_0_ = 80 mM, [PhCO_2_H]_0_ = 400 mM, and [NBS]_0_ = 96 mM. (i) ○ (gray), with [**1**]_0_ = 8 mM. (ii) ◇ (blue), with [**1**]_0_ = 16 mM. (iii) △ (brown), with [**1**]_0_ = 5 mM.

To update Tang et al.’s^3a^ catalytic cycle based on these findings, we note that List has shown strong heterodimerization between BINOL-derived phosphoric acid (*S*)-TRIP and benzoic acid by NMR spectroscopy, with *K*_a_ = 3981 ± 98 M^–1^ in CD_2_Cl_2_.^22^ Hence, we propose herein that the catalyst resting state is heterodimer **4**, which forms through the association of benzoic acid and phosphoric acid **1**, and is particularly likely in our case where the benzoic acid is present in excess (Figure 10). The sodium phosphate salt of **1** does not catalyze the reaction, so catalysis must proceed by reversible proton transfer to NBS (**5**), thereby activating NBS toward reversible electrophilic addition with cyclohexene to form succinimide and to release putative bromonium ion **8** into the electrostatically charge-neutral bulk solution. The weak association of a second carboxylic acid to phosphate **6**—favored by an increase in carboxylic acid concentration—would facilitate subsequent formation of bromoester **2** in a chiral catalyst environment and close the cycle to heterodimer **4**. Alternatively, bromoalkylation of phosphate **6** would give deactivated products **3a** and **3b**.^23^

**Figure 10:**
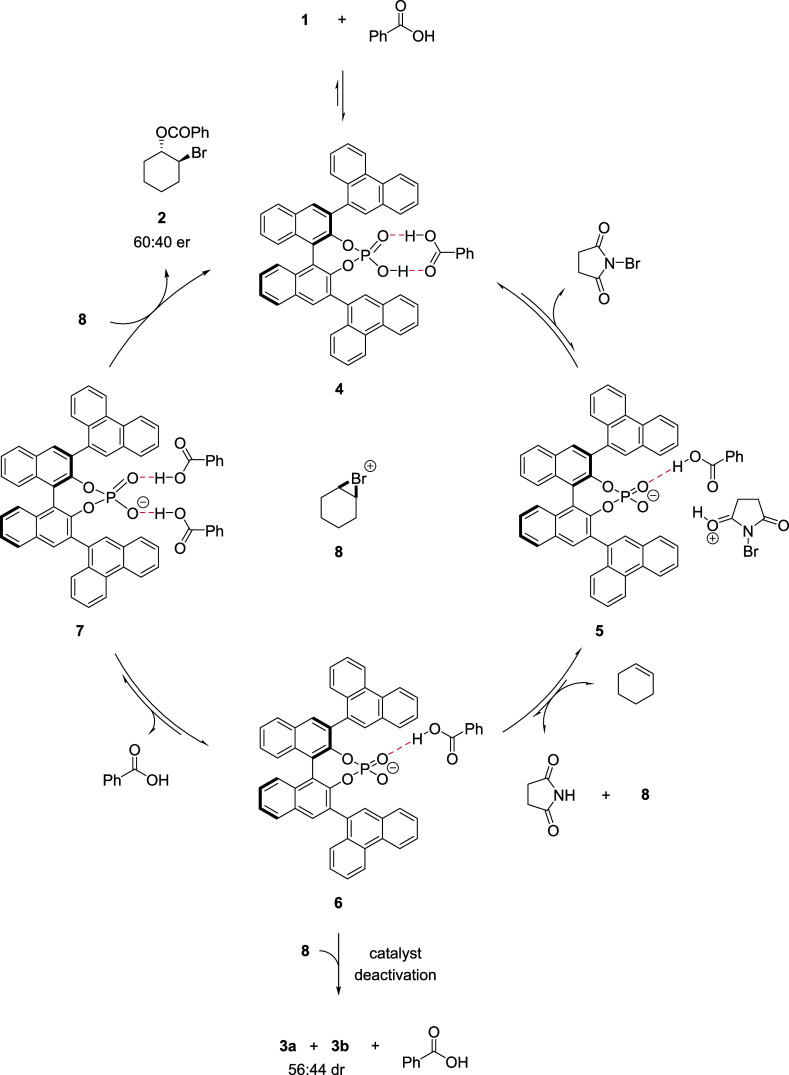
Proposed mechanism for the bromoesterification of cyclohexene.

## Conclusions

In conclusion, we have demonstrated that BINOL-derived chiral phosphoric acid (*R*)-**1** deactivates to bromoalkylated phosphates **3a** and **3b** during the catalytic bromoesterification of cyclohexene. These phosphates were synthesized authentically in an experiment in which the benzoic acid nucleophile was absent. This constitutes the first characterized example of an alkene bromophosphatation process, wherein the phosphate of **1** takes the role of a nucleophile to a putative bromonium ion.^22c,24^ ^31^P­{^1^H} NMR and DFT studies revealed that both phosphates **3a** and **3b** exhibit rotational isomerism with respect to 180-degree rotations about their 9-phenanthrene (Ar) bearing C3,3′–Ar bonds. X-ray diffraction studies were conducted on phosphate **3a** after preparative HPLC separation from **3b** and provided proof of absolute and relative configuration. VTNA studies determined that the catalytic bromoesterification of cyclohexene is first order in all of the reacting components and catalyst **1**, and an updated catalytic cycle has been presented. It is expected that these studies will aid the design of new chiral phosphoric acid derivatives which circumvent deactivation in enantioselective alkene halofunctionalization reactions.^25^

## Experimental Section

### General Experimental Methods

NBS was recrystallized from H_2_O before use, and cyclohexene was distilled before use. Phosphoric acid (*R*)-**1** was prepared following our recommended procedure and was acidified with aqueous 6 M HCl before use.^4a^ All other reagents and commercial grade solvents were utilized without further purification. Flash chromatography used Geduran Si 60, particle size 40–63 μm. Thin layer chromatography (TLC) used Merck Kieselgel 60 F_254_ precoated aluminum-backed plates. The developed plates were visualized by irradiation with UV light (254 or 366 nm) or staining with a KMnO_4_ solution.

^1^H, ^13^C­{^1^H}, and ^31^P­{^1^H} NMR spectra were recorded by using a Bruker AV-400 NMR spectrometer. All chemical shifts (δ) are expressed in ppm (parts per million) relative to the residual solvent peak. Abbreviations for multiplicities are s, singlet; d, doublet; t, triplet; m, multiplet; app, apparent. Fourier transform infrared (IR) spectra were recorded neat on an ATR-IR spectrometer. Mass spectra were recorded by the Imperial College Department of Chemistry Mass Spectroscopy Service. Melting points were recorded using an OptiMelt MPA100 apparatus. Optical rotations were measured on an ADP 440+ polarimeter with a path length of 0.5 dm using the D-line of sodium; concentrations (*c*) are provided in g/100 mL. X-ray crystallography of bromoalkylated phosphate **3a** was conducted by the Imperial College Department of Chemistry X-ray Crystallography Facility with an Agilent Xcalibur PX Ultra A diffractometer. HPLC separations were performed using PerkinElmer Series 200 or Agilent 1260 Infinity II HPLC systems, and the enantiopurity of bromoester **2** was established by HPLC following comparison to an authentic racemic sample. All kinetic bromoesterification experiments were conducted twice or more, and the resulting profiles were averaged between runs.

### Standard Procedure for Cyclohexene Bromoesterification

NBS (51.3 mg, 0.288 mmol) and phosphoric acid **1** (16.8 mg, 0.0240 mmol) were added to an oven-dried, single-necked flask. PhCO_2_H (2.80 mL, 0.429 M in CH_2_Cl_2_, 1.20 mmol) and (4-Tol)_2_CO (0.200 mL, 0.600 M in CH_2_Cl_2_, 0.120 mmol) were injected in the flask, and the solution was stirred at 350 rpm until homogeneous. The solution was cooled to 0 °C and after 10 min, cyclohexene (24.3 μL, 0.240 mmol) was added to begin the experiment. The reaction mixture was sampled (25 μL) at the specified time, and the solution was transferred to a vial containing saturated aqueous Na_2_S_2_O_3_ (200 μL) and MeCN (200 μL). The biphasic mixture was shaken for 30 s, and the top layer was removed and filtered over a cotton packed pipet containing silica gel (∼120 mg) into a HPLC vial. MeCN (300 μL) was eluted through silica into the HPLC vial. [**2**], [**3a**+**3b**], and dr (**3a**+**3b**) were determined by HPLC (SUPELCOSIL LC-18), 50–40–10% H_2_O in MeCN, 1.0 mL/min, λ = 230 nm, *R*_t_ ((4-Tol)_2_CO) = 6.8 min, *R*_t_ (**2**) = 8.7 min, *R*_t_ (**3a**) = 22.5 min, *R*_t_ (**3b**) = 24.0 min. The lids of the vials were unscrewed, and the solvent was left to evaporate. The resulting residue was redissolved in 5% IPA in *n*-hexane (1 mL). er (**2**) was determined by HPLC (CHIRALPAK-AD), 0.5% IPA in *n*-hexane, 1.0 mL/min, λ = 230 nm, *R*_t_ (1*S*,2*S*)-**2** = 9.1 min, and *R*_t_ (1*R*,2*R*)-**2** = 10.1 min. Other experiments where the initial concentrations of catalyst **1**, NBS, cyclohexene, or PhCO_2_H were varied were conducted by using the above procedure with adjustments to the stoichiometry as specified.

### Same Excess Procedure for Cyclohexene Bromoesterification

NBS (40.6 mg, 0.228 mmol) and phosphoric acid **1** (16.8 mg, 0.0240 mmol) were added to an oven-dried, single-necked flask. CH_2_Cl_2_ (0.140 mL) **or** bromoester *rac*-**2** (0.140 mL, 0.429 M in CH_2_Cl_2_, 0.060 mmol), then PhCO_2_H (2.66 mL, 0.429 M in CH_2_Cl_2_, 1.14 mmol) **or** PhCO_2_H + succinimide (2.66 mL, 0.429 M + 0.0226 M in CH_2_Cl_2_, 1.14 mmol + 0.0600 mmol) and (4-Tol)_2_CO (0.200 mL, 0.600 M in CH_2_Cl_2_, 0.120 mmol) were injected in the flask, and the solution was stirred at 350 rpm until homogeneous. The solution was cooled to 0 °C, and after 10 min, cyclohexene (18.2 μL, 0.180 mmol) was added. The reaction mixture was sampled and analyzed as above.

#### (1*S*,2*S*)-(+)-2-Bromocyclohexyl Benzoate (**2**)

NBS (51.3 mg, 0.288 mmol) and phosphoric acid **1** (16.8 mg, 0.0240 mmol) were added to an oven-dried, single-necked flask. PhCO_2_H (2.80 mL, 0.429 M in CH_2_Cl_2_, 1.20 mmol) and CH_2_Cl_2_ (0.200 mL) were injected in the flask, and the solution was stirred at 350 rpm until homogeneous. The solution was cooled to 0 °C and after 10 min, cyclohexene (24.3 μL, 0.240 mmol) was added. After 8 h, the reaction mixture was washed with saturated aqueous Na_2_S_2_O_3_ (5 mL), the layers were separated, and the aqueous phase was extracted with CH_2_Cl_2_ (3 × 5 mL). The combined organics were dried over MgSO_4_, filtered and concentrated *in vacuo*. The resulting residue was chromatographed (3% EtOAc in petroleum ether) to provide bromoester **2** (17.8 mg, 26%) as a colorless oil. *R*_f_ = 0.40 (3% EtOAc in petroleum ether); ${[\alpha ]}_{D}^{24}$ = +20.2 (*c* = 0.89, CHCl_3_) (lit.^26^ ${[\alpha ]}_{D}^{24}$ = +104.6, *c* = 1.0, CHCl_3_, 95:5 er); IR (film) 1712 cm^–1^; ^1^H NMR (400 MHz, CDCl_3_) δ 8.09–8.06 (m, 2H), 7.57 (app t, *J* = 7.6 Hz, 1H), 7.45 (app t, *J* = 7.6 Hz, 2H), 5.13 (ddd, *J* = 9.2, 9.1, 4.4 Hz, 1H), 4.16 (ddd, *J* = 10.4, 9.1, 4.4 Hz, 1H), 2.45–2.38 (m, 1H), 2.32–2.23 (m, 1H), 1.95 (dddd, *J* = 17.1, 11.2, 7.3, 3.9 Hz, 1H), 1.86–1.74 (m, 2H), 1.58–1.34 (m, 3H); ^13^C­{^1^H} NMR (101 MHz, CDCl_3_) δ 165.7, 133.2, 130.4, 129.8, 128.5, 76.5, 52.8, 35.7, 31.2, 25.5, 23.4; HRMS (APCI^+^, ion trap) calcd. for C_13_H_15_O_2_ (M–Br)^+^, 203.1067; found, 203.1059; HPLC (CHIRALPAK-AD), 0.5% IPA in *n*-hexane, 1.0 mL/min, λ = 230 nm, *R*_t_ (1*S*,2*S*)-**2** = 9.1 min, and *R*_t_ (1*R*,2*R*)-**2** = 10.1 min (59:41 er).

### Bromophosphatation of Cyclohexene

#### Bromocyclohexyl Phosphate (R,1R,2R)-**3a** and Bromocyclohexyl Phosphate (R,1S,2S)-**3b**

Cyclohexene (4.1 μL, 0.040 mmol) was added to a stirred solution of NBS (8.5 mg, 0.048 mmol) and phosphoric acid **1** (28.0 mg, 0.0400 mmol) in CH_2_Cl_2_ (0.5 mL) at 0 °C. After 2.5 h, the cloudy reaction mixture was directly chromatographed (CH_2_Cl_2_) to provide phosphates **3a**+**3b** (16.7 mg, 48%) as a white solid. *R*_f_ = 0.80 (CH_2_Cl_2_); mp. 200 °C (dec.); IR (film) 1295 cm^–1^; ^1^H NMR (400 MHz, acetone-*d*_6_) δ 8.95–8.79 (m, 4H), 8.42–8.13 (m, 4H), 8.08–7.91 (m, 4H), 7.90–7.40 (m, 16H), 3.64–3.48 (m, 0.74H), 3.42–3.19 (m, 0.73H), 3.16–3.10 (m, 0.32H), 2.74–2.68 (m, 0.16H), 2.28–2.11 (m, 0.23H), 1.81–0.21 (m, 8.49H), −0.97––1.08 (m, 0.18H); ^13^C­{^1^H} NMR (101 MHz, acetone-*d*_6_) δ 206.1, 147.1, 145.9, 135.4, 135.1, 134.6, 134.4, 134.3, 134.2, 134.0, 133.6, 133.49, 133.45, 133.4, 133.2, 133.0, 132.8, 132.6, 132.50, 132.45, 132.1, 132.0, 131.9, 131.8, 131.43, 131.36, 131.3, 131.2, 131.02, 130.97, 130.8, 130.7, 130.4, 130.2, 130.1, 129.9, 129.74, 129.68, 129.64, 129.59, 128.3, 128.12, 128.07, 128.02, 127.98, 127.93, 127.87, 127.83, 127.79, 127.7, 127.64, 127.55, 127.5, 127.4, 127.32, 127.25, 127.2, 124.0, 123.82, 123.76, 123.7, 123.6, 123.52, 123.46, 123.4, 123.0, 122.4, 122.2, 83.5, 83.1, 82.1, 52.2, 51.7, 32.1, 31.2, 25.3, 24.8, 23.1, 22.9, 21.9; ^31^P­{^1^H} NMR (162 MHz, acetone-*d*_6_) δ 0.1 (**3a**), 0.0 (**3a**), −0.2 (**3a**), −0.3 (**3a**), −0.6 (**3b**), −0.7 (**3b**), −1.2 (**3b**), −1.3 (**3b**) (multiple signals due to the presence of rotamers); HRMS (ESI^+^, TOF) calcd. for C_54_H_39_^79^BrO_4_P (M+H)^+^, 861.1764; found, 861.1760; HPLC (SUPELCOSIL LC-18), 10% H_2_O in MeCN, 1.0 mL/min, λ = 230 nm, *R*_t_ (**3a**) = 9.1 min, *R*_t_ (**3b**) = 10.5 min (56:44 dr); HPLC (CHIRALPAK-AD), 20% IPA in *n*-hexane, 1.0 mL/min, λ = 230 nm, *R*_t_ (**3a**) = 6.9 min, *R*_t_ (**3b**) = 9.2 min (both diastereoisomers >99:1 er). HPLC was used to preparatively separate the **3a** and **3b** diastereoisomers, providing pure phosphates **3a** (5.5 mg) and **3b** (3.9 mg, 91:9 dr) as white solids, after 20 runs. HPLC (Poroshell 120 SB-C18), 100% MeCN, 25 mL/min, λ = 250 nm, 400 μL injection, 3 mg/mL sample concentration in MeCN, *R*_t_ (**3a**) = 3.7 min, and *R*_t_ (**3b**) = 3.9 min. Crystal data for **3a**: C_54_H_38_BrO_4_P·2.25­(C_6_H_12_), *M* = 1051.07, orthorhombic, *P*2_1_2_1_2_1_ (no. 19), *a* = 8.4319(3), *b* = 20.6529(6), *c* = 31.3293(8) Å, *V* = 5455.8(3) Å^3^, *Z* = 4, *D*_c_ = 1.280 g cm^–3^, μ­(Mo–Kα) = 0.833 mm^–1^, *T* = 173 K, colorless tablets, Agilent Xcalibur 3 E diffractometer; 10,763 independent measured reflections (*R*_int_ = 0.0264), *F* ^2^ refinement,^27^ *R*_1_(obs) = 0.0476, *wR*_2_(all) = 0.1066, 8210 independent observed absorption-corrected reflections [|*F*_0_| > 4σ­(|*F*_0_|), completeness to θ_full_(25.2°) = 99.4%], 541 parameters. The absolute configuration of phosphate **3a** was determined by the use of the Flack parameter [*x* ^+^ = 0.007(4)]. CCDC 2395133.

## Supplementary Material



## Data Availability

The data underlying this study are available in the published article, in its Supporting Information, and openly available in the Imperial College research data repository at 10.14469/hpc/14708.

## Notes

HPLC calibration method; representative HPLC chromatograms; different excess experiments; X-ray structure of phosphate **3a**; calculation of Boltzmann populations; variable temperature ^31^P­{^1^H} NMR experiment; and copies of ^1^H, ^13^C­{^1^H}, and ^31^P­{^1^H} NMR spectra (PDF)
