# Supplementary material for: Bromophosphatation as a Mode of Chiral Phosphoric Acid Catalyst Deactivation as Elucidated by Kinetic Profiling
Source: J Org Chem. 2025 May 20;90(21):6992–7002. doi: 10.1021/acs.joc.5c00431 (PMC12131226; doi:10.1021/acs.joc.5c00431)
Supplement: Supplementary file 1 [file jo5c00431_si_001.pdf]

Electronic Supporting Information

Bromophosphatation as a Mode of Chiral Phosphoric Acid Catalyst Deactivation as Elucidated by Kinetic Profiling

Ben M. J. Lancaster,[a] Andrew J. P. White,[a] Christopher J. Tighe,*[b] and D. Christopher Braddock*[a]

[a] Department of Chemistry, Imperial College London, Molecular Sciences Research Hub, White City Campus, 82 Wood Lane, London W12 0BZ, UK

[b] Department of Chemical Engineering, Imperial College London, South Kensington Campus, Imperial College Road, London SW7 2AZ, UK

* E-mail: c.braddock@imperial.ac.uk, c.tighe@imperial.ac.uk

Cover Page and Contents

Cover Page and Contents: S1

1. HPLC Calibration Method; S2–S3

2. Representative HPLC Chromatograms; S4–S5

3. Different Excess Experiments; S6–S12

4. X-Ray Structure of Phosphate **3a**; S13

5. Calculation of Boltzmann Populations; S14

6. Variable Temperature $^{31}P\{^{1}H\}$ NMR Experiment; S15

7. Copies of ^{1}H, $^{13}C\{^{1}H\}$ and $^{31}P\{^{1}H\}$ NMR Spectra; S16–S24

8. References. S25

1. HPLC Calibration Method

1.1. [2] Calibration

[**2**] was calculated as described in Equation S1 using the HPLC peak area of bromoester **2** relative to the area of $(4\text{-Tol})_2CO$ (which was constant during catalysis).

$$[\mathbf{2}] = \frac{\text{HPLC area } (\mathbf{2})}{\text{HPLC area } (4\text{-Tol})_2\text{CO}} \cdot \frac{[(4\text{-Tol})_2\text{CO}]_0}{m} \qquad \text{(S1)}$$

where $[(4\text{-Tol})_2CO]_0$ = 40 mM for all experiments, and *m* is the gradient derived from Figure S1.

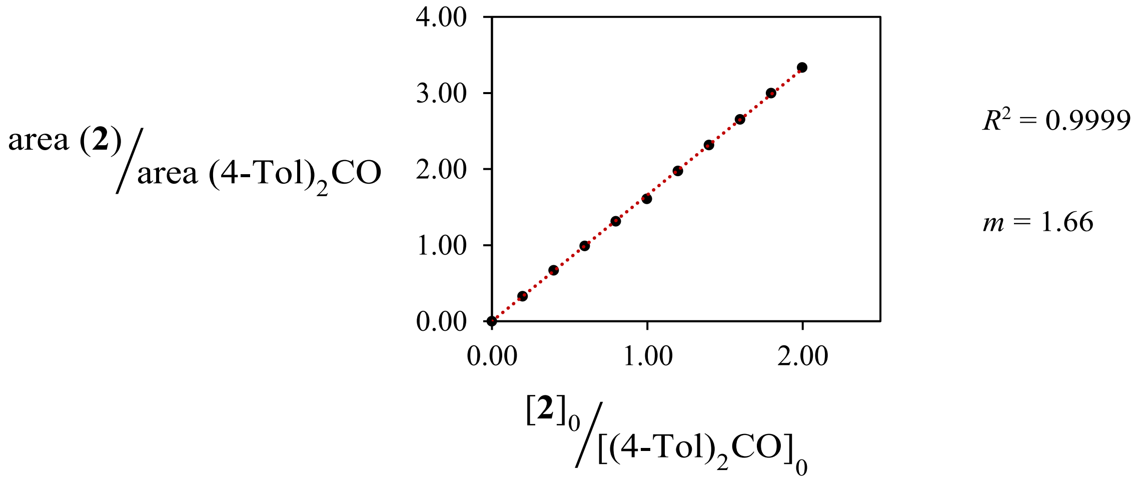

Figure S1. HPLC calibration of bromoester *rac*-**2** and $(4\text{-Tol})_2CO$.

HPLC (SUPELCOSIL LC-18), 50−40−10% H_2O in MeCN, 1.0 mL/min,

λ = 230 nm, R_t ($(4\text{-Tol})_2CO$) = 6.8 min, R_t (**2**) = 8.7 min.

1.2. [3a+3b] Calibration

[**3a**+**3b**] was calculated as described in Equation S2 using the HPLC peak area of phosphates **3a** and **3b** relative to the area of $(4\text{-Tol})_2CO$.

$$[\mathbf{3a}+\mathbf{3b}] = \frac{\text{HPLC area }(\mathbf{3a}+\mathbf{3b})}{\text{HPLC area }(4\text{-Tol})_2\text{CO}} \cdot \frac{[(4\text{-Tol})_2\text{CO}]_0}{m} \tag{S2}$$

where $[(4\text{-Tol})_2CO]_0$ = 40 mM for all experiments. $^{31}P\{^1H\}$ NMR measurements were conducted on a select number of HPLC samples that had been evaporated and redissolved in DMSO-d_6, allowing m to be determined as 25.63 by comparison of Equation S2 with Equation S3.

$$[\mathbf{3a}+\mathbf{3b}] = \frac{\text{NMR area }(\mathbf{3a}+\mathbf{3b})}{\text{NMR area }(\mathbf{1}) + \text{NMR area }(\mathbf{3a}+\mathbf{3b})} \cdot [\mathbf{1}]_0 \tag{S3}$$

$$m = \frac{\text{HPLC area }(\mathbf{3a}+\mathbf{3b})}{\text{HPLC area }(4\text{-Tol})_2\text{CO}} \cdot \frac{\text{NMR area }(\mathbf{1}) + \text{NMR area }(\mathbf{3a}+\mathbf{3b})}{\text{NMR area }(\mathbf{3a}+\mathbf{3b})} \cdot \frac{[(4\text{-Tol})_2\text{CO}]_0}{[\mathbf{1}]_0} \tag{S4}$$

2. Representative HPLC Chromatograms

HPLC chromatograms of *rac*- and (1*S*,2*S*)-(+)-2-bromocyclohexyl benzoate (**2**) (59:41 er)

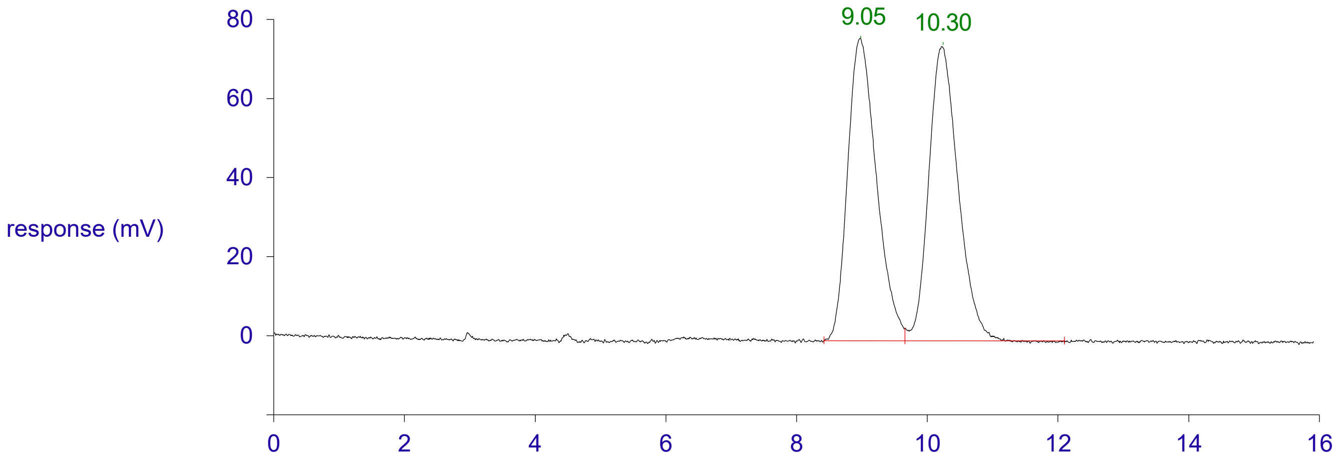

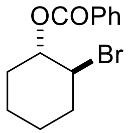

racemate

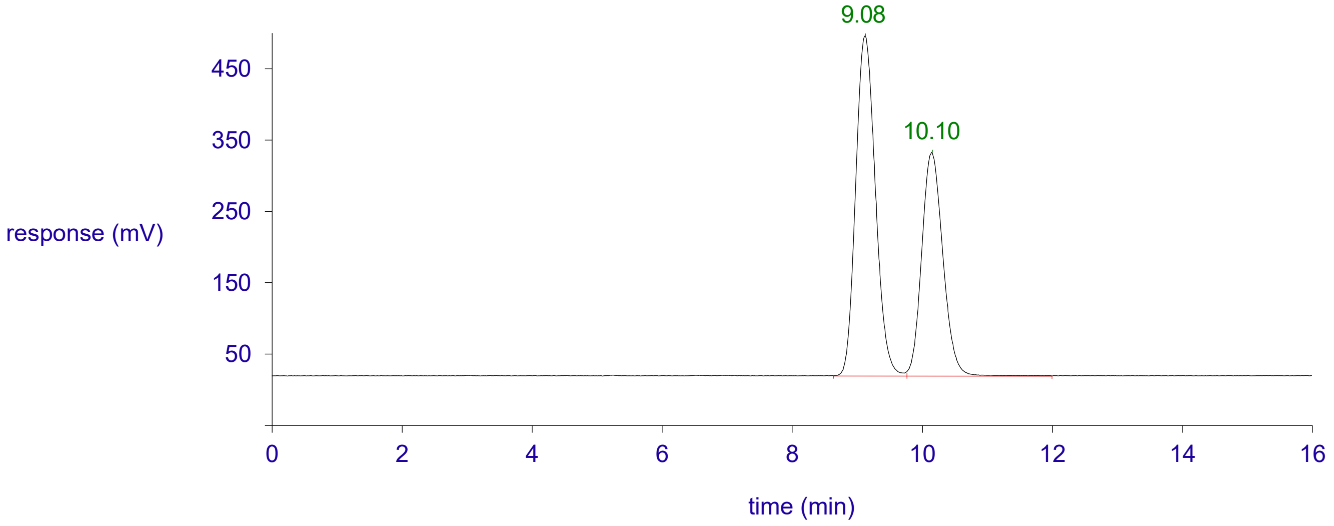

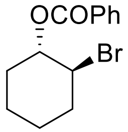

(1*S*,2*S*)-enantiomer

HPLC chromatograms of bromocyclohexyl phosphates **3a**+**3b** (56:44 dr), (*R*,1*R*,2*R*)-**3a** (>99:1 dr) and (*R*,1*S*,2*S*)-**3b** (>99:1 dr)

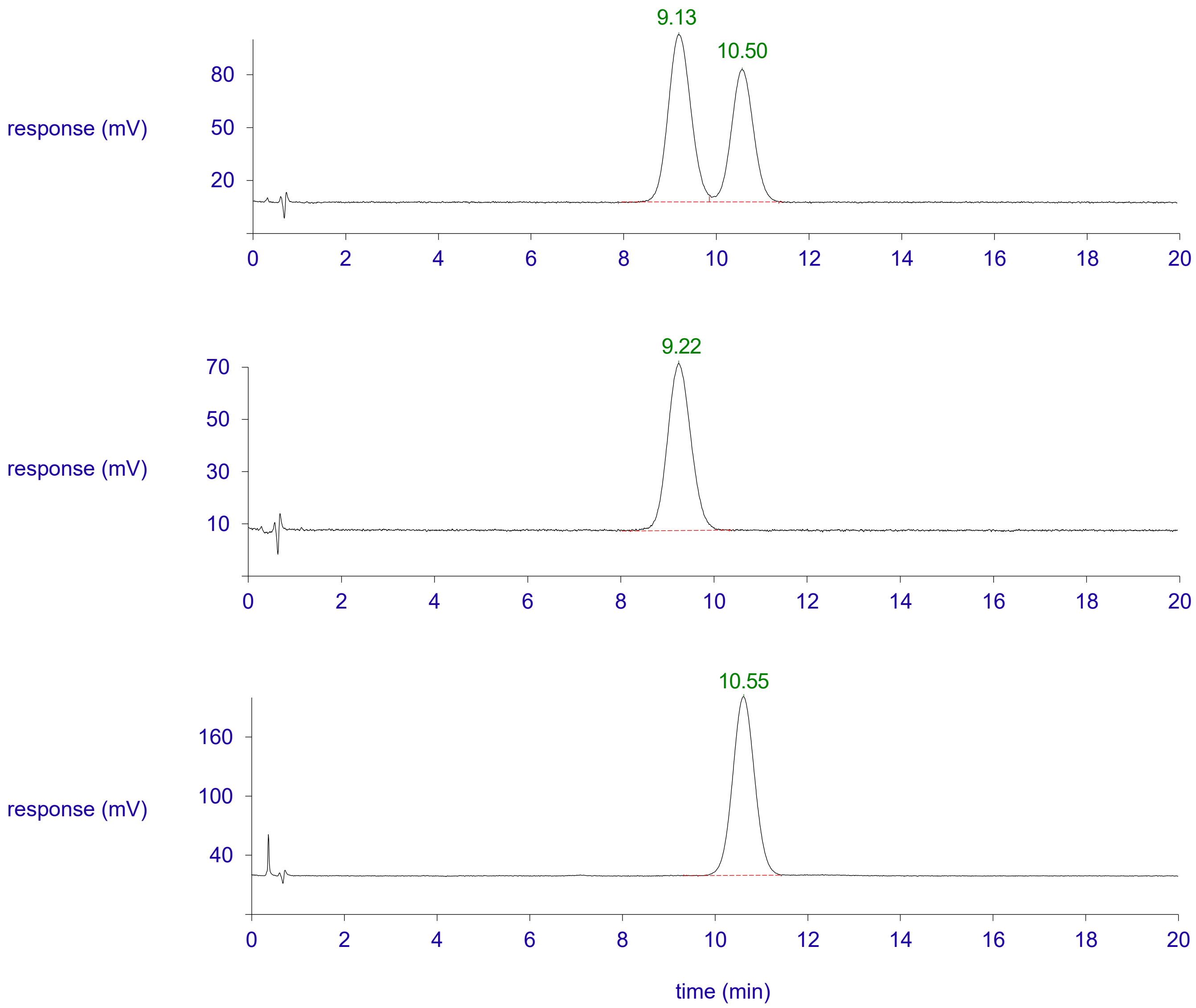

mixture

3a

3b

(the >99:1 dr sample of phosphate **3b** results from repurification of 91:9 dr **3b**; the mass recovered was insufficient for NMR studies).

3. Different Excess Experiments

Further information on different excess experiments not discussed in detail in the manuscript is given below.

3.1. 4,4'-Dimethylbenzophenone

4,4'-Dimethylbenzophenone, the internal standard, was determined to be zeroth order due to overlay of the profiles without normalization of the timescale (Figure S2).

Table S1. Tabulated initial concentrations in experiments of varying $(4\text{-Tol})_2CO$ concentration.

concentration (mM)	standard	varied
$[\text{cyclohexene}]_0$	80	80
$[PhCO_2H]_0$	400	400
$[\text{NBS}]_0$	96	96
$[\mathbf{1}]_0$	8	8
$[(4\text{-Tol})_2CO]_0$	40	20

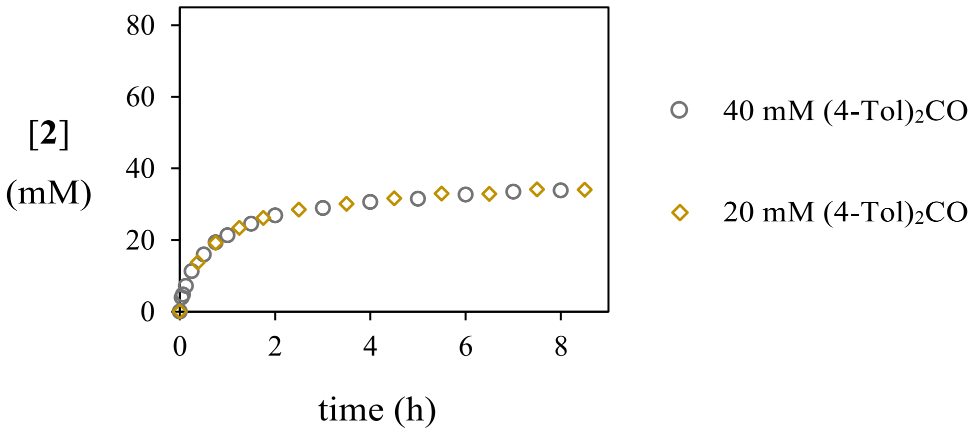

Figure S2. Plot of [**2**] *vs* time in experiments of varying $(4\text{-Tol})_2CO$ concentration (Table S1) as monitored by HPLC methods.

3.2. *N*-Bromosuccinimide

NBS was determined to be first order due to overlay of the profiles (Figure S3b) when the normalized timescale was raised to a power of 1.0.

$$\sum [\mathrm{NBS}]^{\alpha}\Delta t = \sum_{i=1}^{n} \left(\frac{[\mathrm{NBS}]_i + [\mathrm{NBS}]_{i-1}}{2}\right)^{\alpha} \cdot (t_i - t_{i-1}) \qquad \text{(S5)}$$

where [NBS] = $[\mathrm{NBS}]_0$ – [**2**]

Although [NBS] = $[\mathrm{NBS}]_0$ − [**2**] − [**3a**+**3b**], [**2**] >> [**3a**+**3b**], and thus a subtraction of [**3a**+**3b**] (which we did not quantify in this case) was neglected; this treatment also applies to the [cyclohexene] in the next section.

Table S2. Tabulated initial concentrations in experiments of varying NBS concentration.

concentration (mM)	standard	varied
$[\text{cyclohexene}]_0$	80	80
$[\mathrm{PhCO_2H}]_0$	400	400
$[\mathrm{NBS}]_0$	96	64
[**1**]$_0$	8	8
$[(\text{4-Tol})_2\mathrm{CO}]_0$	40	40

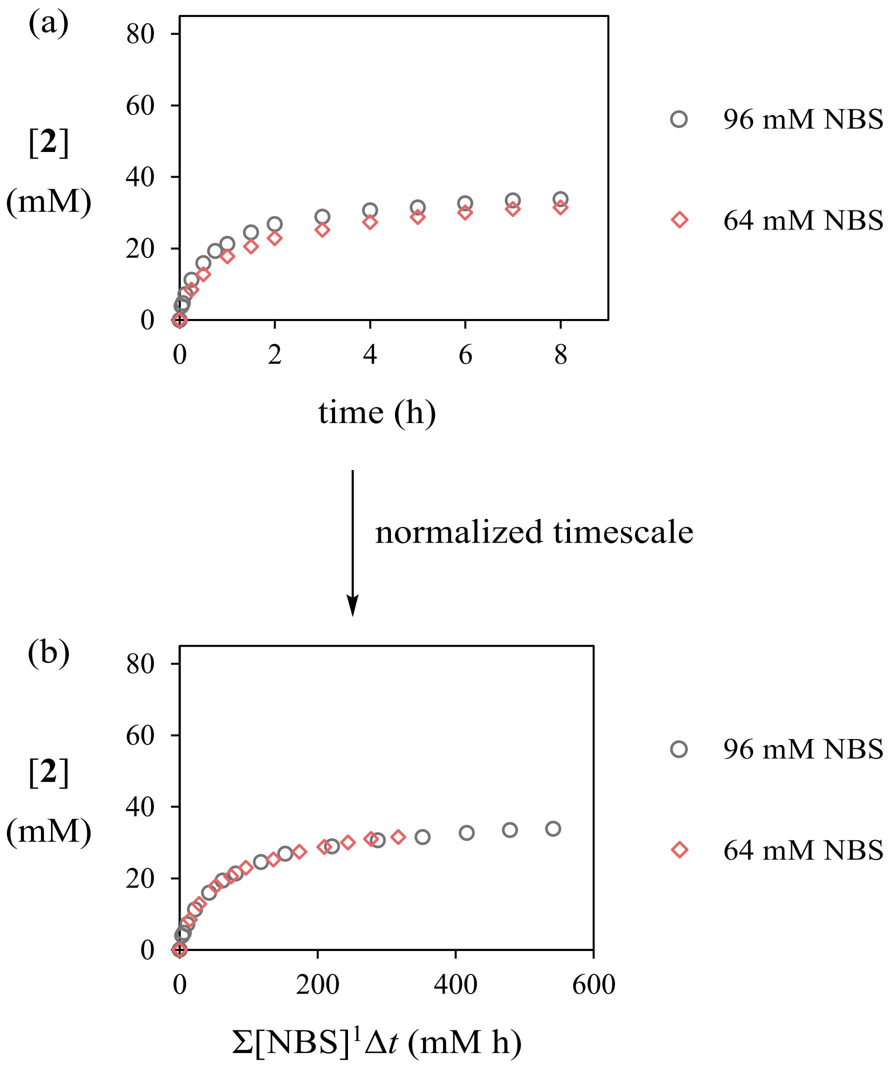

Figure S3. Plots of (a) [**2**] *vs* time and (b) [**2**] *vs* normalized time in experiments of varying NBS concentration (Table S2) as monitored by HPLC methods.

3.3. Cyclohexene

Cyclohexene was determined to be first order due to overlay of the profiles (Figure S4b) when the normalized timescale was raised to a power of 1.0.

$$\sum [\text{cyclohexene}]^{\beta} \Delta t = \sum_{i=1}^{n} \left(\frac{[\text{cyclohexene}]_i + [\text{cyclohexene}]_{i-1}}{2} \right)^{\beta} \cdot (t_i - t_{i-1}) \quad \text{(S6)}$$

where [cyclohexene] = $[\text{cyclohexene}]_0$ – [**2**]

Table S3. Tabulated initial concentrations in experiments of varying cyclohexene concentration.

concentration (mM)	standard	varied
$[\text{cyclohexene}]_0$	80	40
$[\text{PhCO}_2\text{H}]_0$	400	400
$[\text{NBS}]_0$	96	96
$[\mathbf{1}]_0$	8	8
$[(\text{4-Tol})_2\text{CO}]_0$	40	40

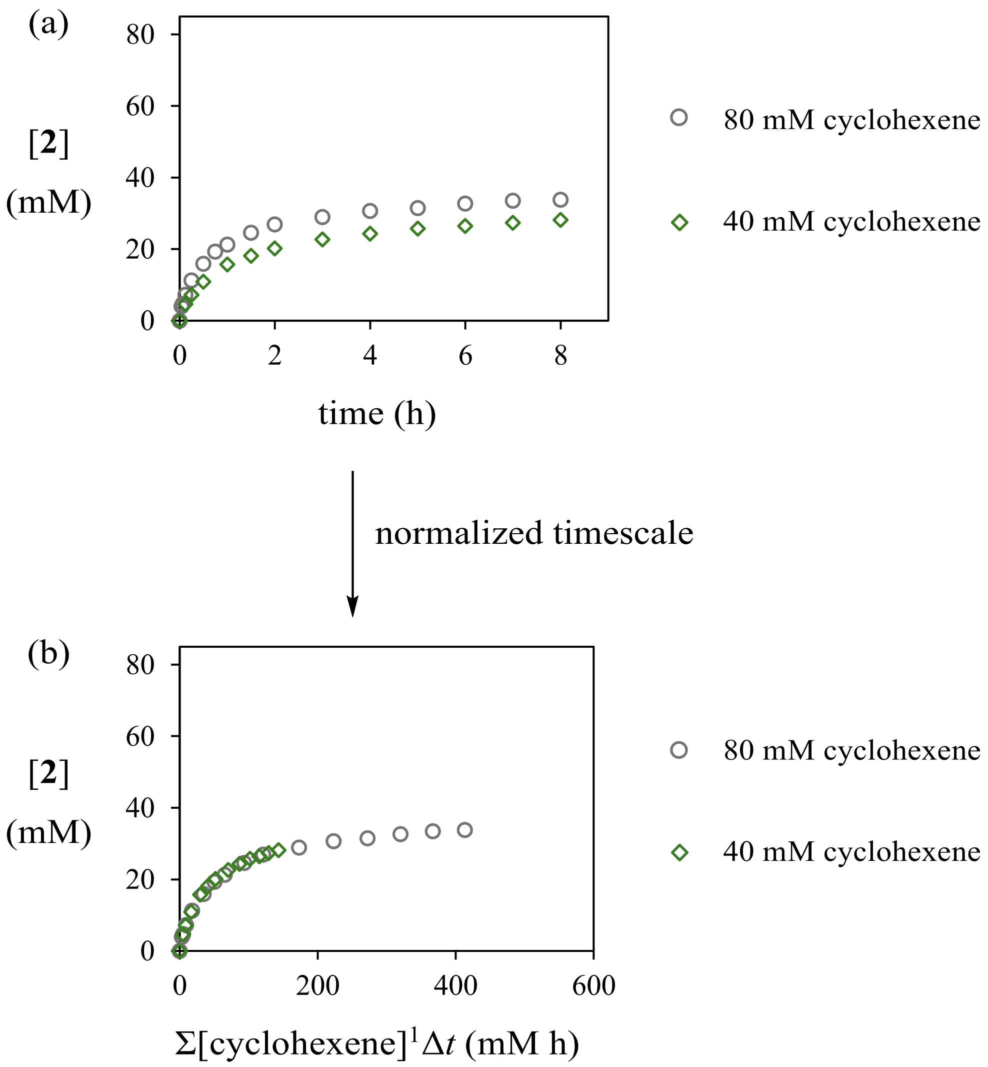

Figure S4. Plots of (a) [**2**] *vs* time and (b) [**2**] *vs* normalized time in experiments of varying cyclohexene concentration (Table S3) as monitored by HPLC methods.

3.4. Benzoic Acid

Benzoic acid was determined to be first order due to overlay of the profiles (at the onset only) for the bromoesterification reaction (Figure S5.1b) when the normalized timescale was raised to a power for 1.0.

Additionally, an order of –0.7 was obtained for the bromophosphatation reaction under catalytic conditions (Figure S5.2b).

$$\sum [\mathrm{PhCO_2H}]^{\delta}\Delta t = \sum_{i=1}^{n} \left(\frac{[\mathrm{PhCO_2H}]_i + [\mathrm{PhCO_2H}]_{i-1}}{2}\right)^{\delta} \cdot (t_i - t_{i-1}) \qquad \text{(S7)}$$

where $[\mathrm{PhCO_2H}] = [\mathrm{PhCO_2H}]_0 - [\mathbf{2}]$

Table S4. Tabulated initial concentrations in experiments of varying benzoic acid concentration.

concentration (mM)	standard		varied	
$[\text{cyclohexene}]_0$	80	80	80	80
$[\mathrm{PhCO_2H}]_0$	400	160	280	650
$[\mathrm{NBS}]_0$	96	96	96	96
$[\mathbf{1}]_0$	8	8	8	8
$[(\text{4-Tol})_2\mathrm{CO}]_0$	40	40	40	40

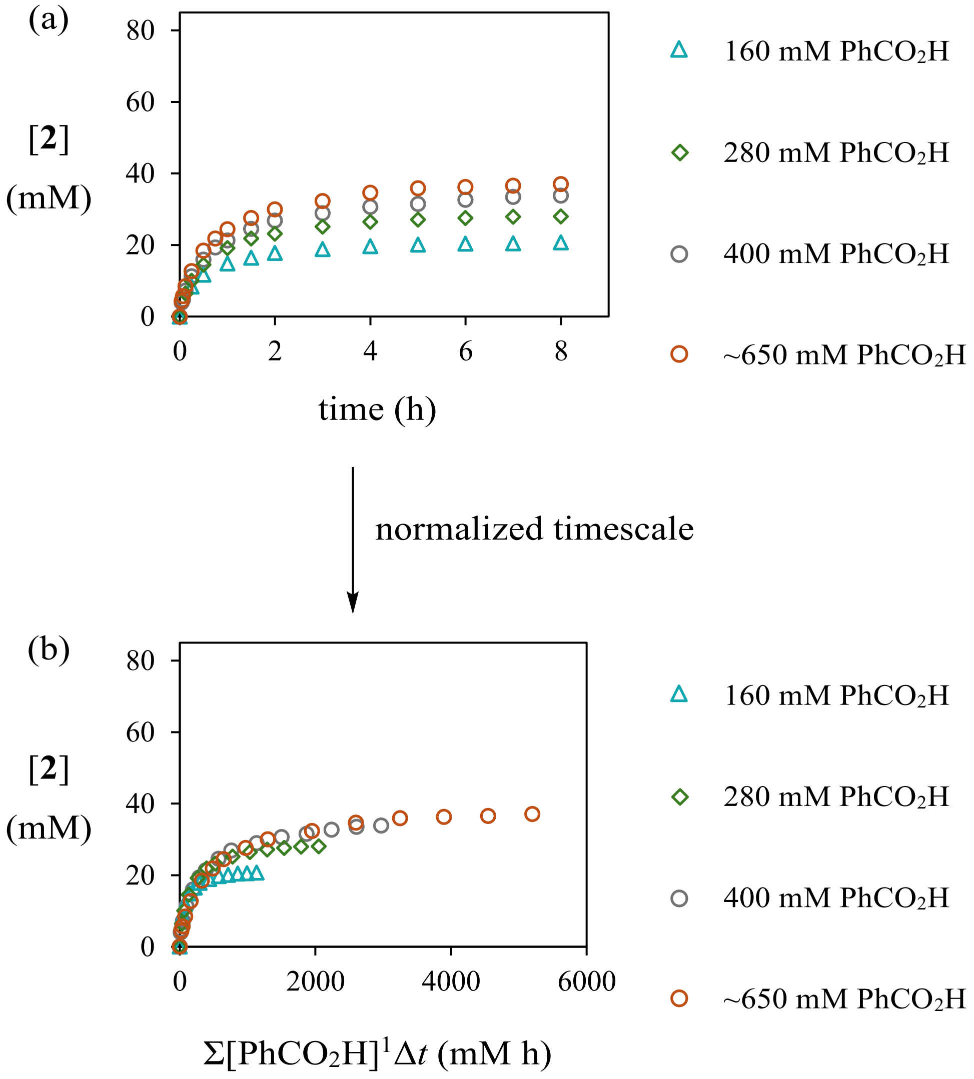

Figure S5.1. Plots of (a) [**2**] *vs* time and (b) [**2**] *vs* normalized time in experiments of varying benzoic acid concentration (Table S4) as monitored by HPLC methods.

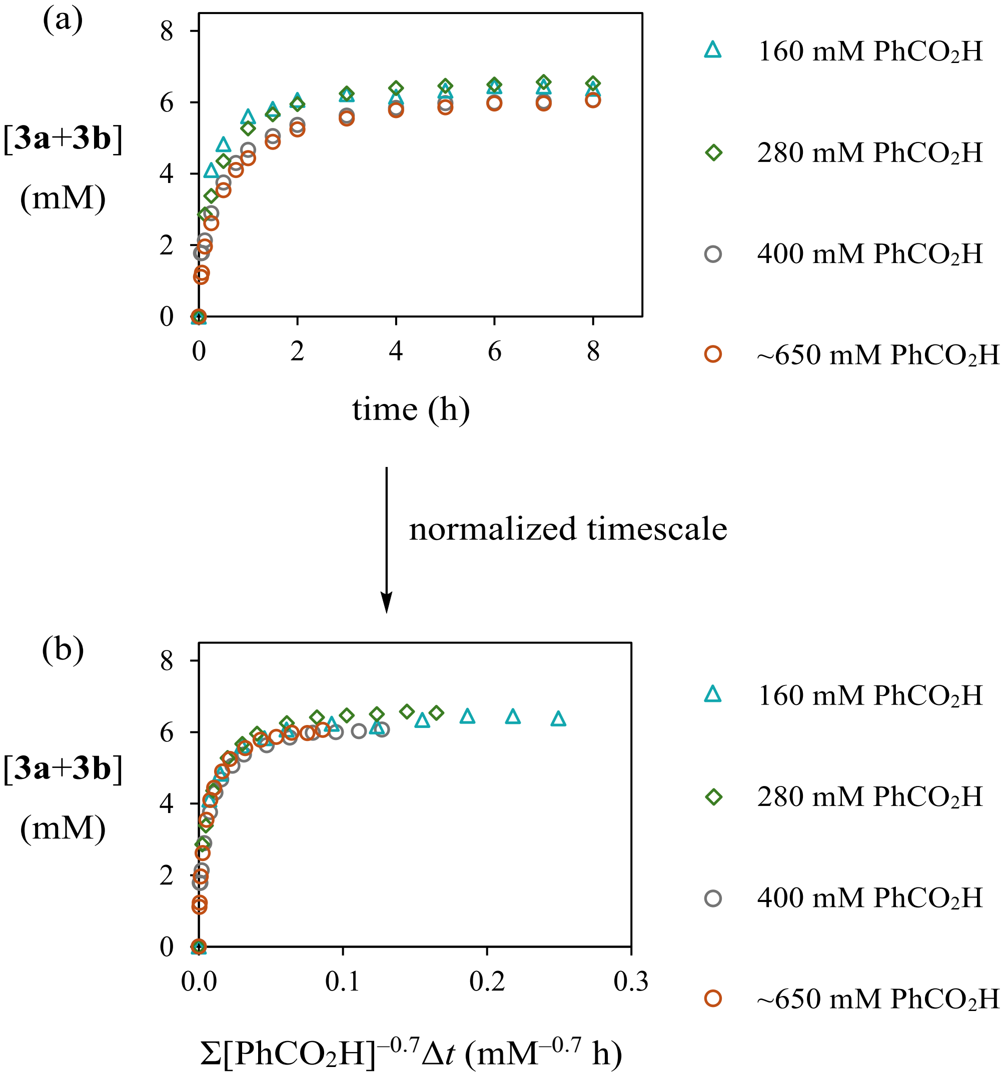

Figure S5.2. Plots of (a) [**3a**+**3b**] *vs* time and (b) [**3a**+**3b**] *vs* normalized time in experiments of varying benzoic acid concentration (Table S4) as monitored by HPLC methods.

4. X-Ray Structure of Phosphate 3a

The included solvent in the structure of **3a** was found to be highly disordered, and the best approach to handling this diffuse electron density was found to be the SQUEEZE routine of PLATON.[1] This suggested a total of 429 electrons per unit cell, equivalent to 107.3 electrons per asymmetric unit. Before the use of SQUEEZE the solvent most resembled cyclohexane (C_6H_{12}, 48 electrons), and 2.25 cyclohexane molecules corresponds to 108 electrons, so this was used as the solvent present. Therefore, the atom list for the asymmetric unit is low by 2.25(C_6H_{12}) = $C_{13.5}H_{27}$ (and that for the unit cell low by $C_{54}H_{108}$) compared to what is actually presumed to be present.

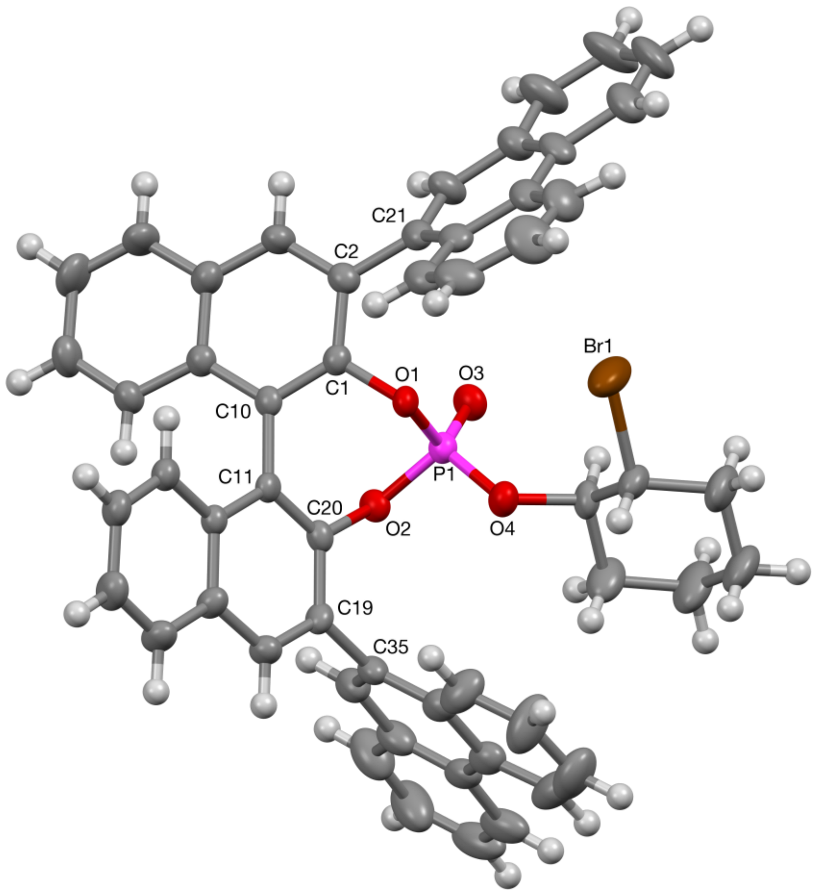

Figure S6. X-ray crystal structure of phosphate **3a** (50% probability ellipsoids).

5. Calculation of Boltzmann Populations

Boltzmann populations were computed at 298 K as described in Equation S8 using the DFT optimized potential energies of four rotational isomers of **3a** (calculated using the B3LYP/ def2-SVP level of theory and the IEFPCM solvation method in acetone).

$$\frac{n_i}{n} = \frac{\exp(-E_i/RT)}{\sum_{i=1}^{4} \exp(-E_i/RT)} \tag{S8}$$

where n_i/n is the population of each rotamer in proportion to all of the modeled rotamers, E_i is the DFT calculated potential energy of each rotamer in J/mol, R is the ideal gas constant, and T is the temperature.

6. Variable Temperature $^{31}P\{^{1}H\}$ NMR Experiment

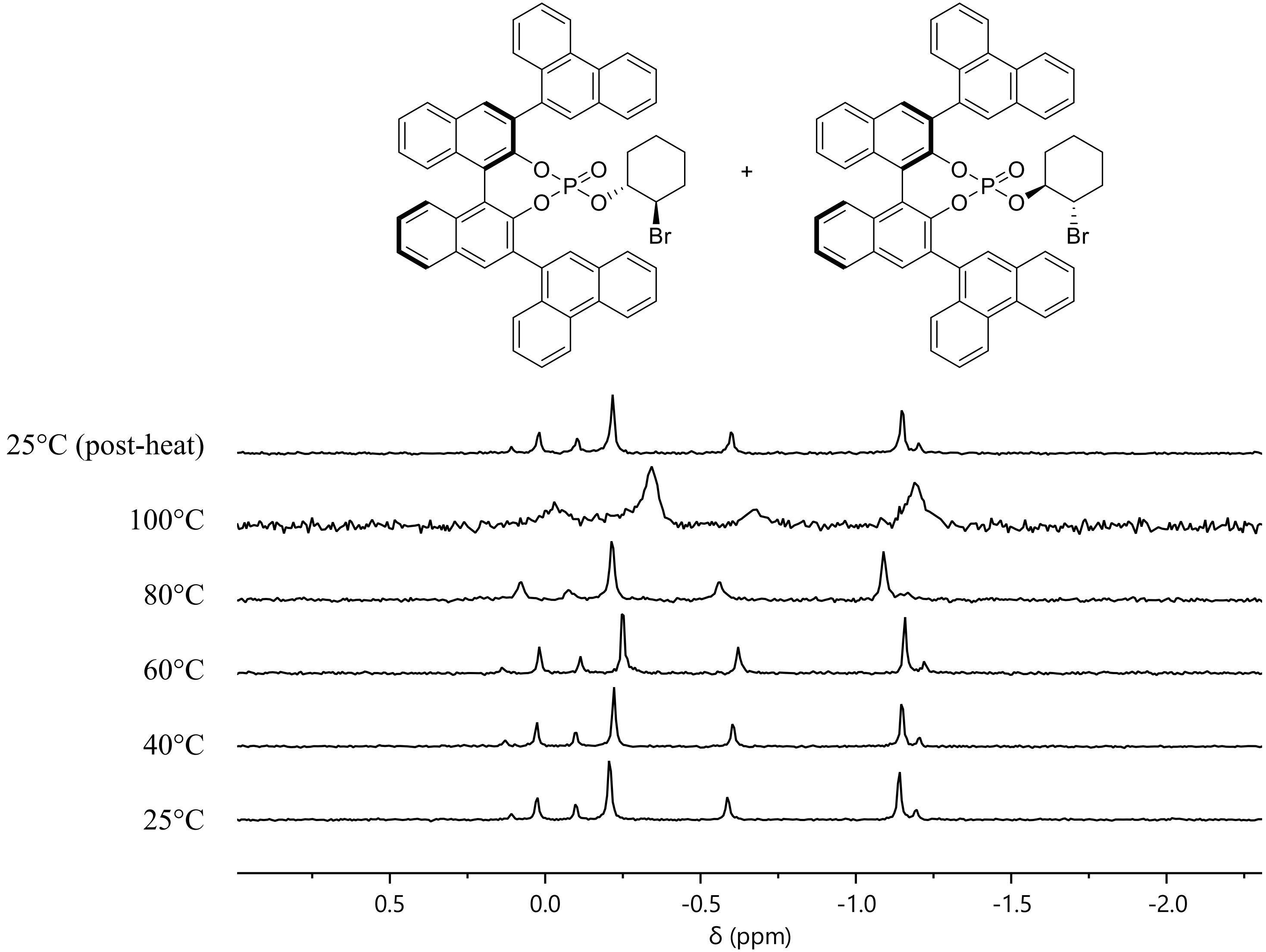

Figure S7. VT $^{31}P\{^{1}H\}$ NMR spectra of bromoalkylated phosphates **3a** and **3b** (56:44 dr) in DMSO-d_6.

7. Copies of ^{1}H, ^{13}C{^{1}H} and ^{31}P{^{1}H} NMR Spectra

^{1}H NMR spectrum of (1*S*,2*S*)-(+)-2-bromocyclohexyl benzoate (**2**) (60:40 er) (400 MHz, $CDCl_3$)

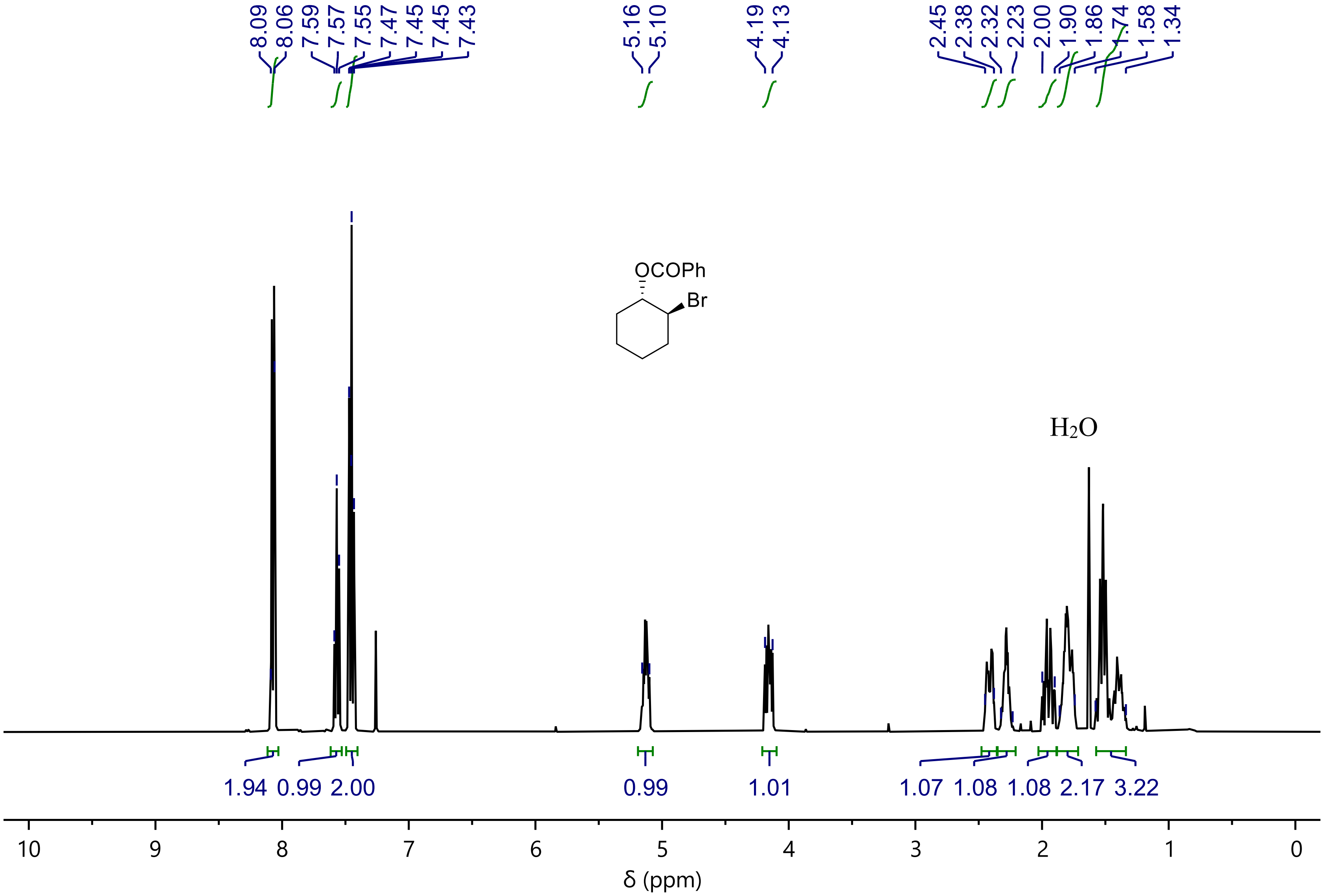

$^{13}C\{^1H\}$ NMR spectrum of (1*S*,2*S*)-(+)-2-bromocyclohexyl benzoate (**2**) (60:40 er) (101 MHz, $CDCl_3$)

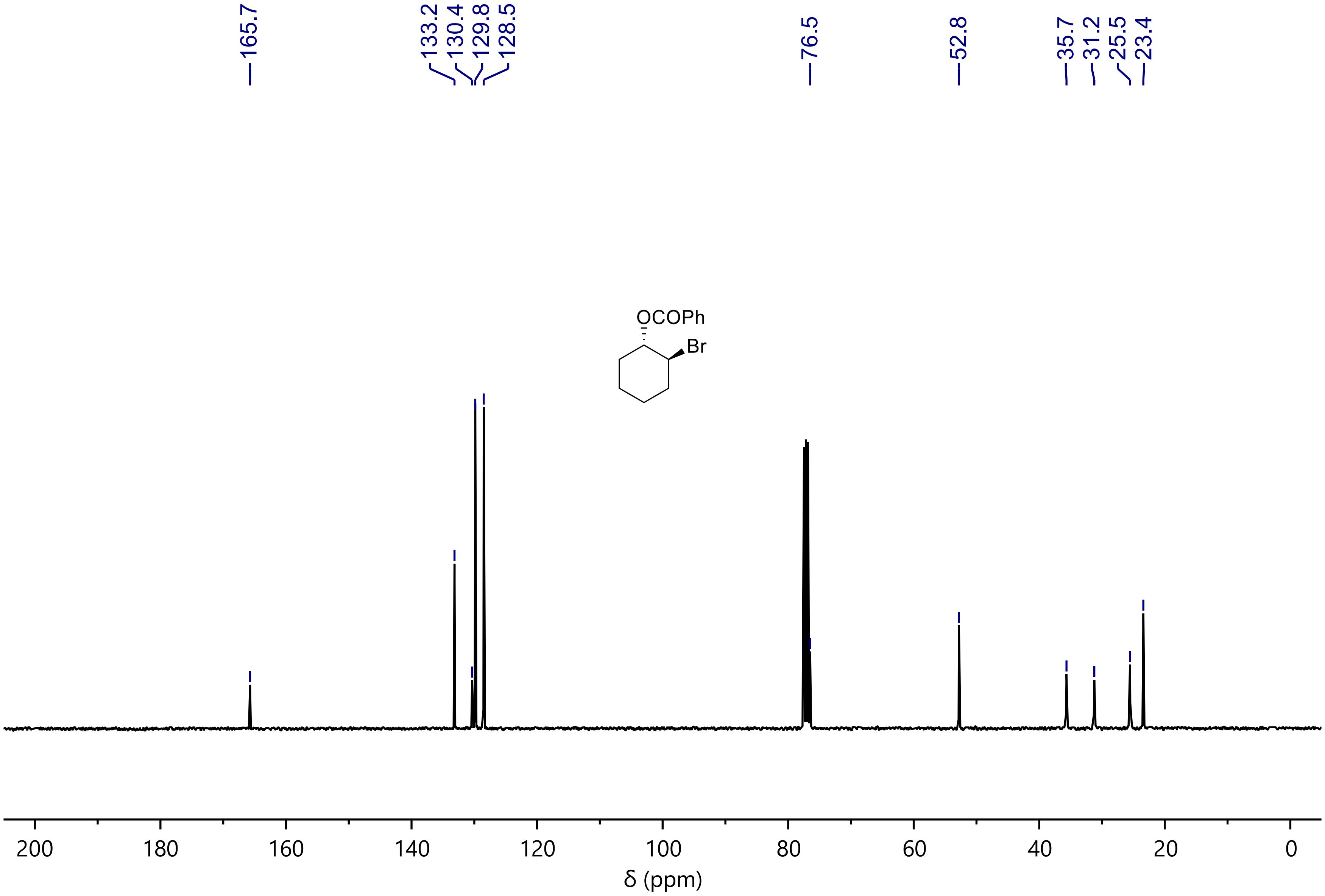

^{1}H NMR spectrum of bromocyclohexyl phosphates **3a**+**3b** (56:44 dr) (400 MHz, acetone-d_6)

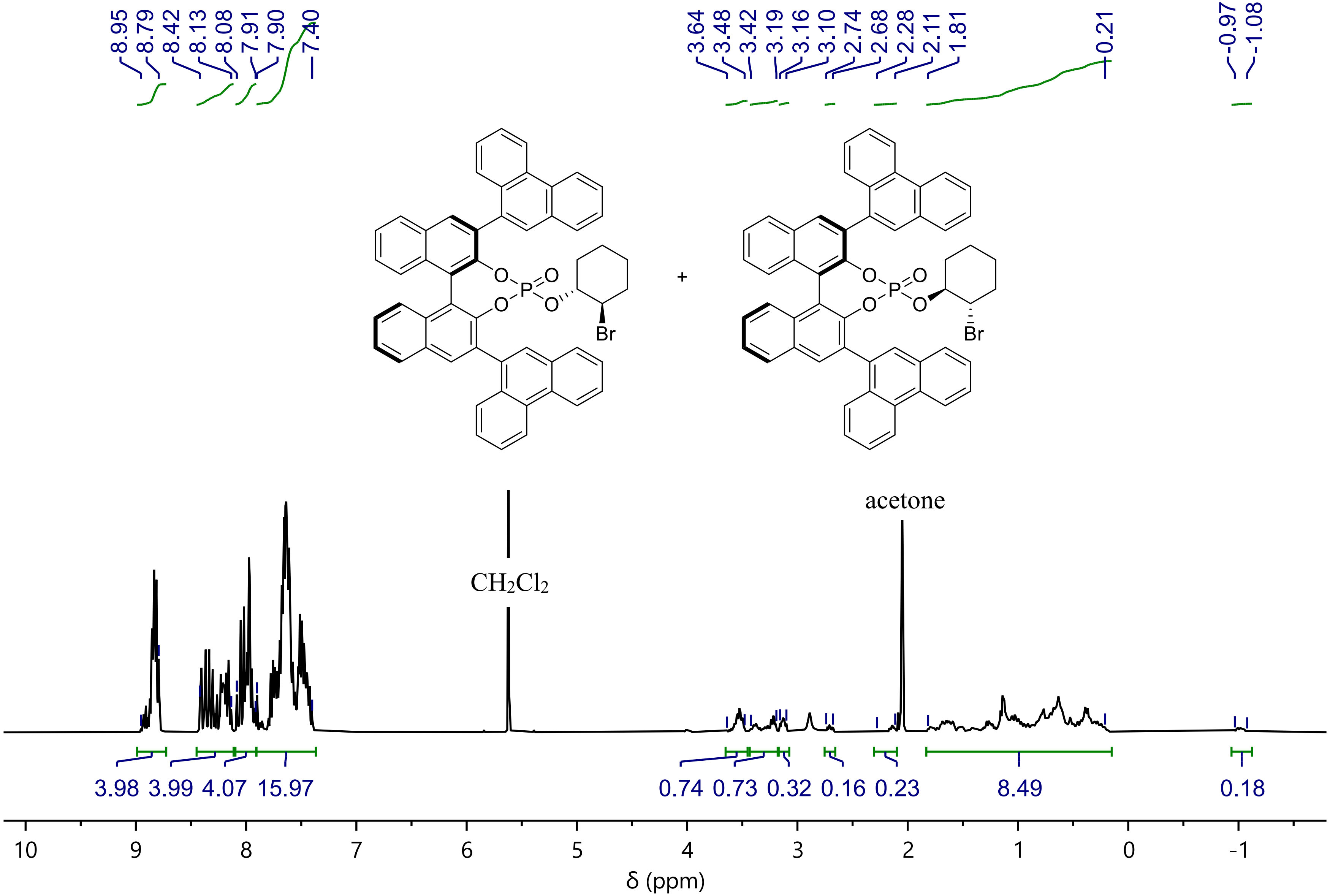

$^{13}C\{^{1}H\}$ NMR spectrum of bromocyclohexyl phosphates **3a**+**3b** (56:44 dr) (101 MHz, acetone-d_6)

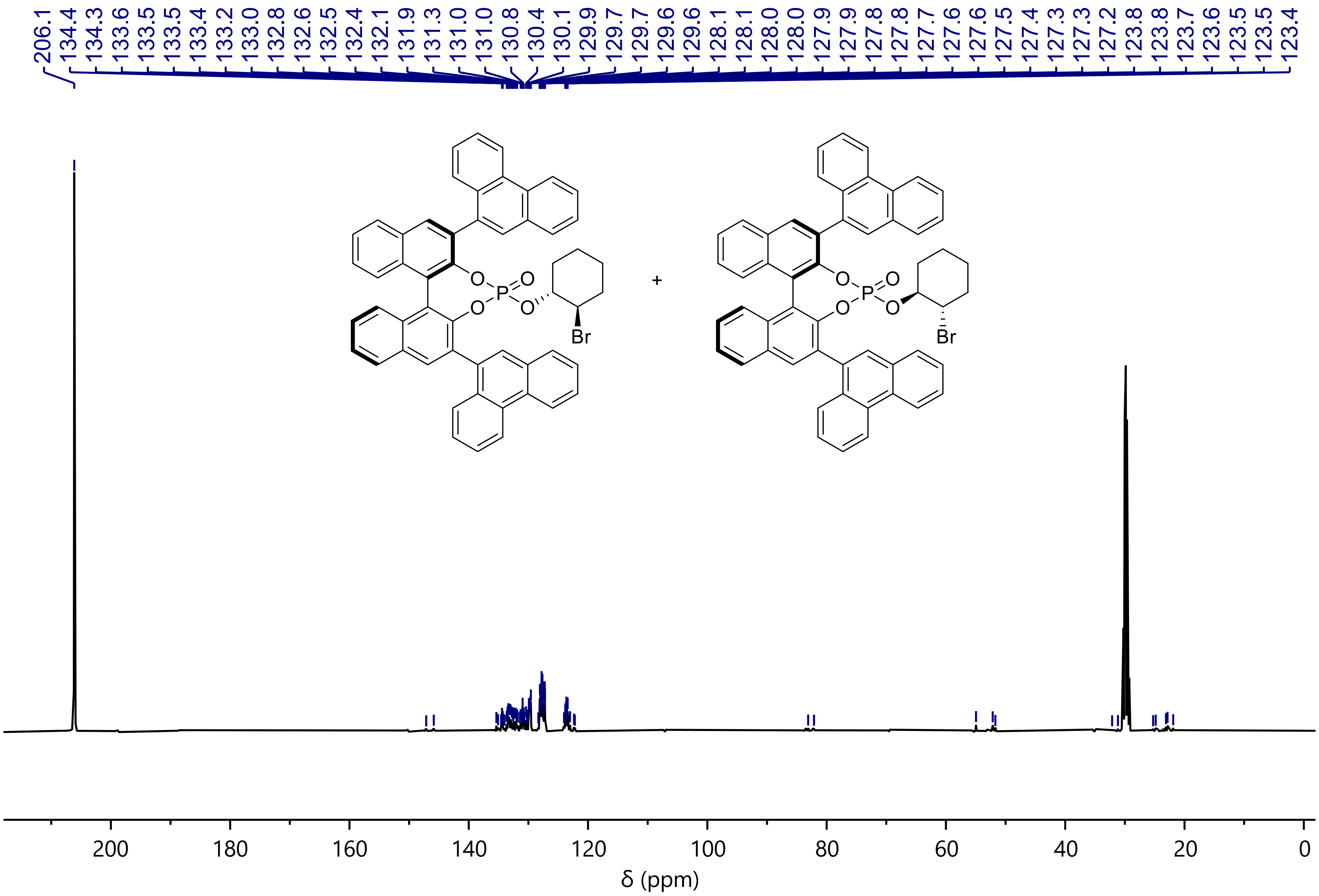

$^{31}P\{^{1}H\}$ NMR spectrum of bromocyclohexyl phosphates **3a+3b** (56:44 dr) (162 MHz, acetone-d_6)

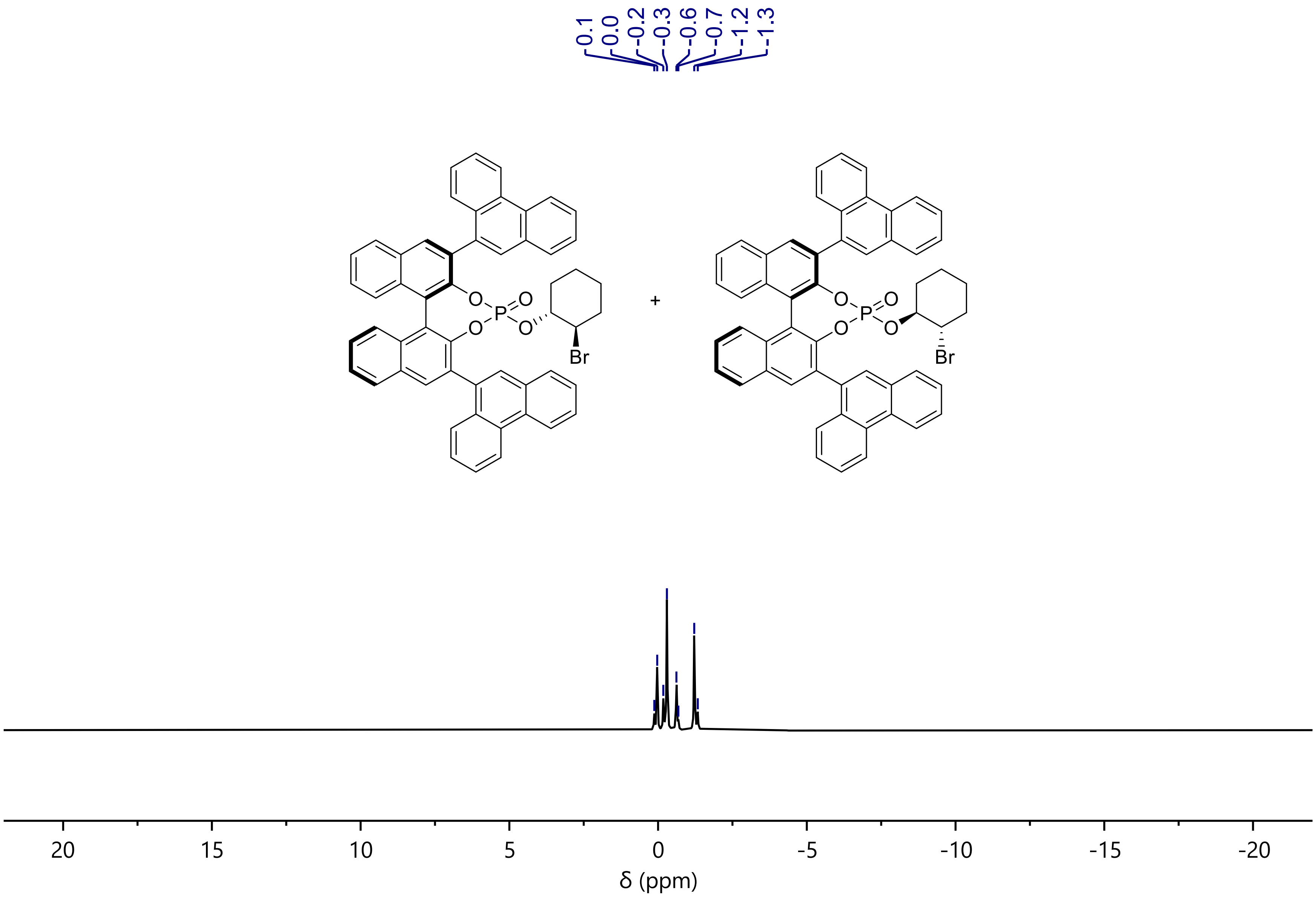

^{1}H NMR spectrum of bromocyclohexyl phosphate **3a** (>99:1 dr) (400 MHz, acetone-d_6)

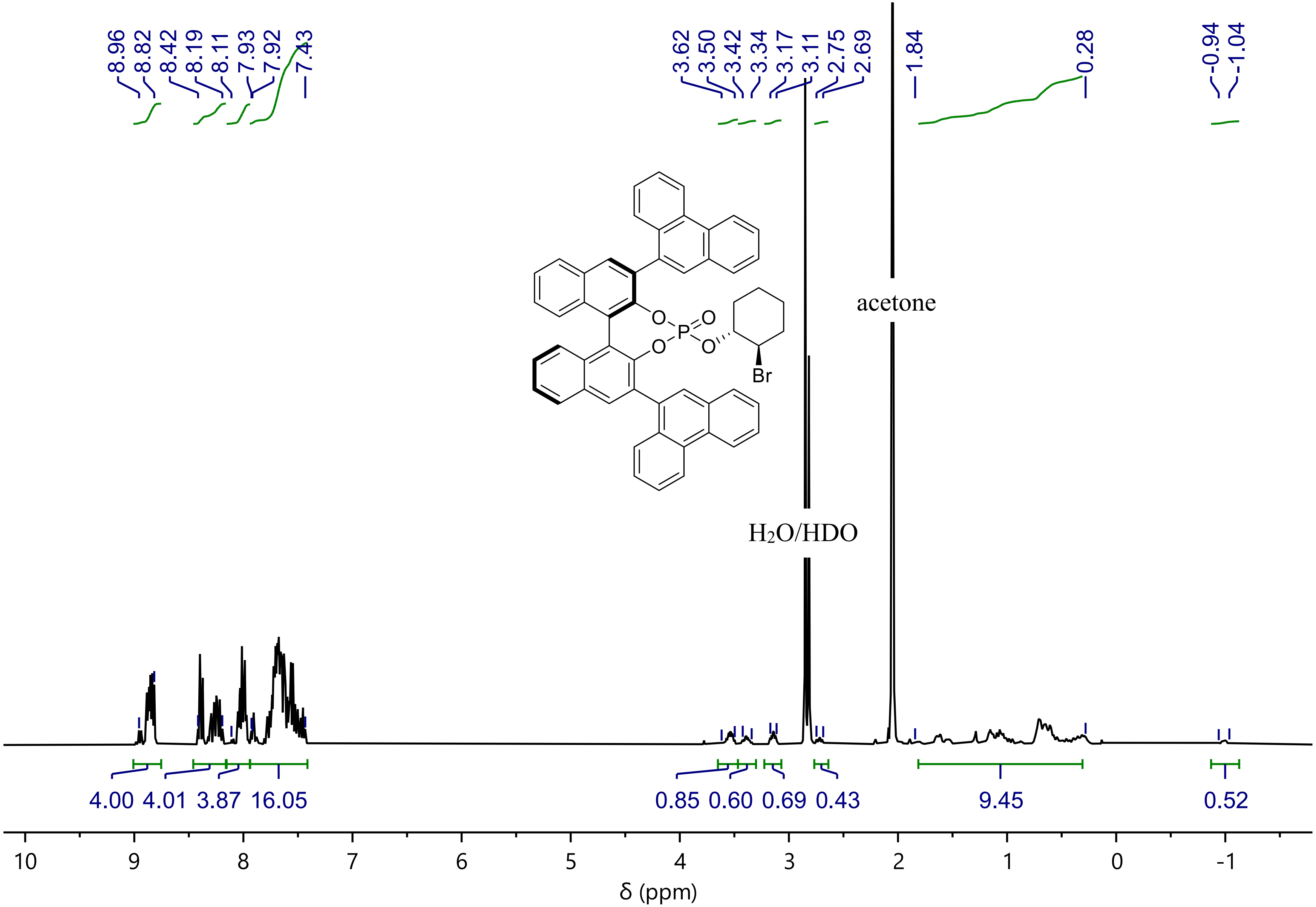

$^{31}P\{^{1}H\}$ NMR spectrum of bromocyclohexyl phosphate **3a** (>99:1 dr) (162 MHz, acetone-*d*$_6$)

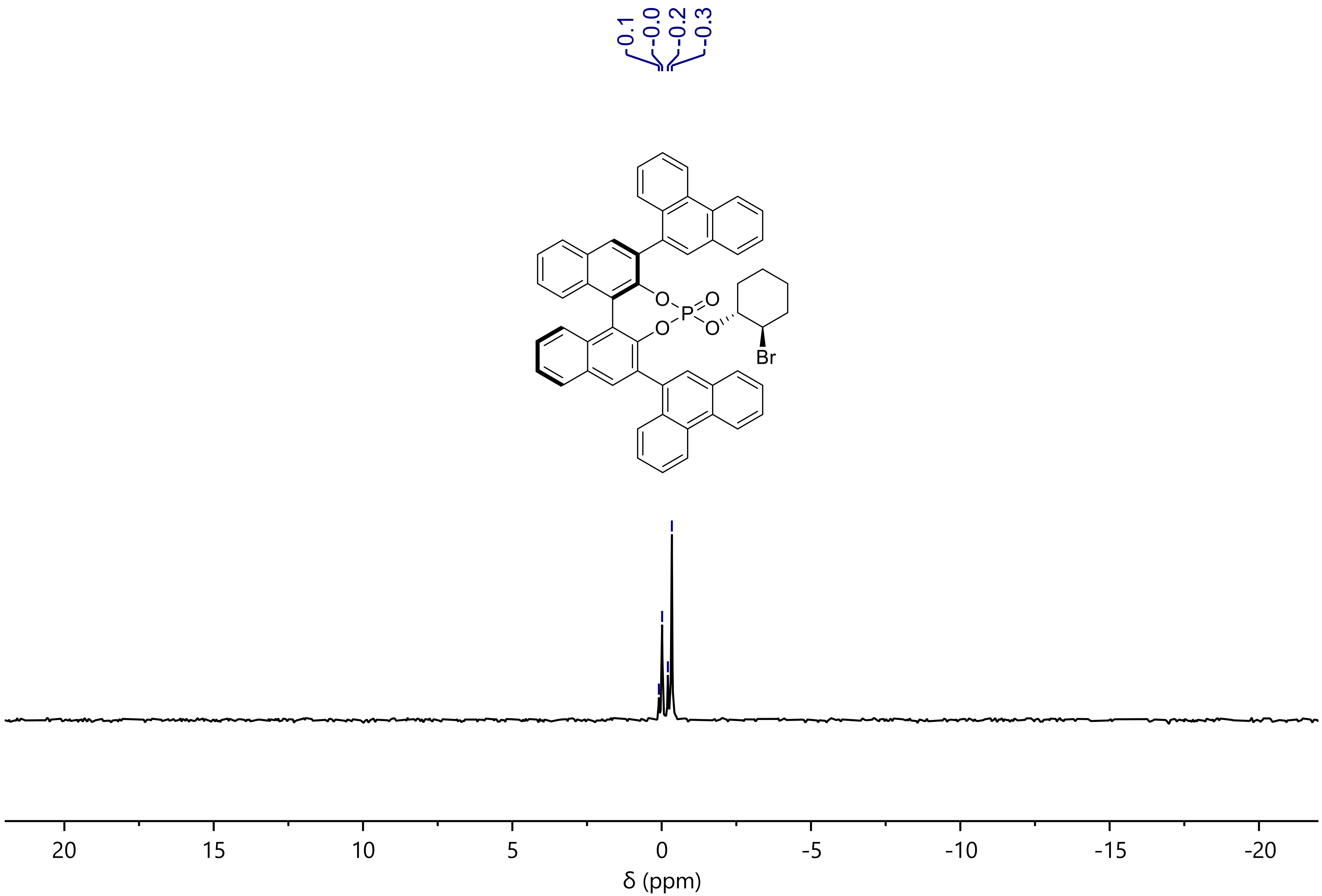

^{1}H NMR spectrum of bromocyclohexyl phosphate **3b** (91:9 dr) (400 MHz, acetone-d_6)

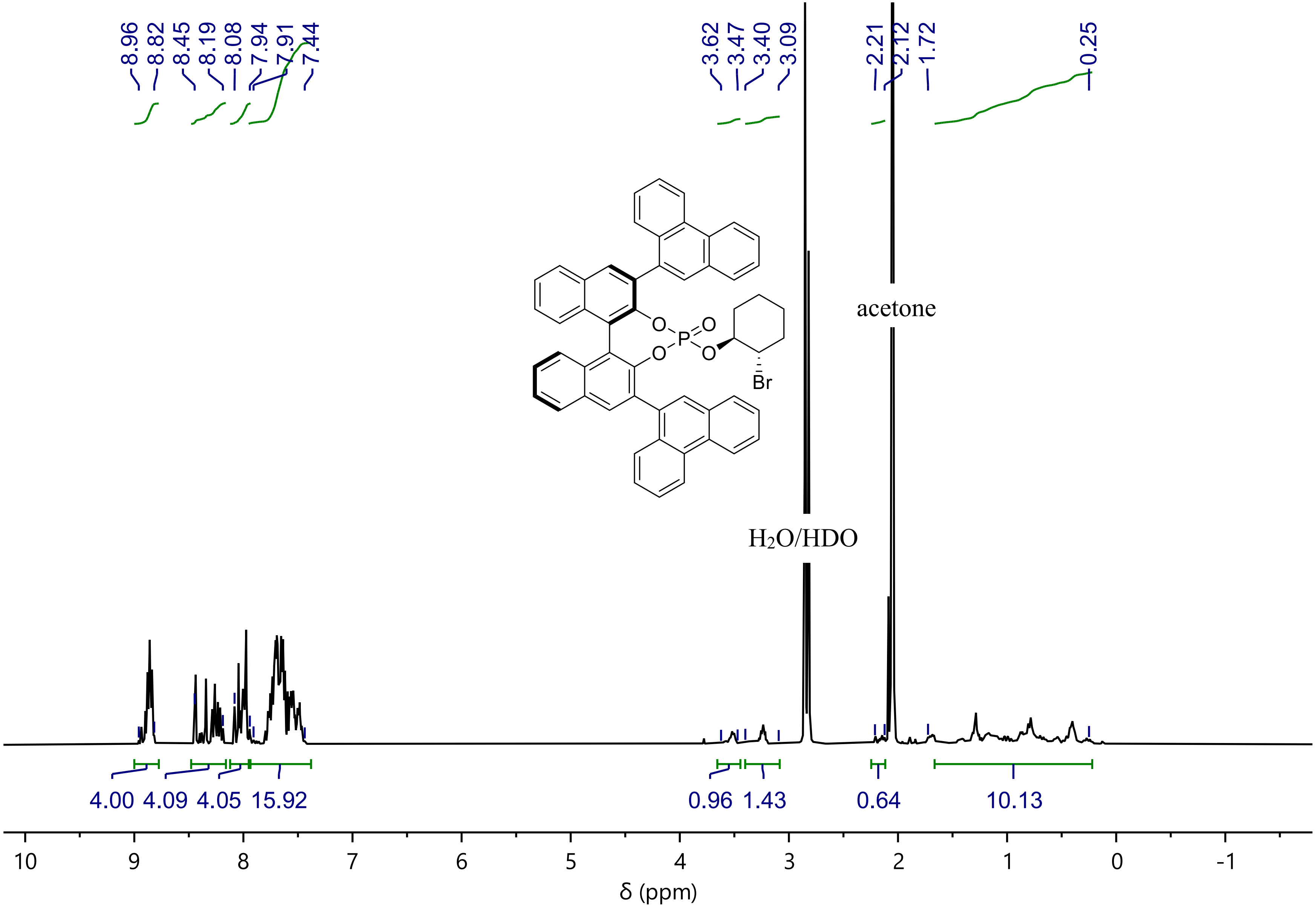

$^{31}P\{^1H\}$ NMR spectrum of bromocyclohexyl phosphate **3b** (91:9 dr) (162 MHz, acetone-d_6)

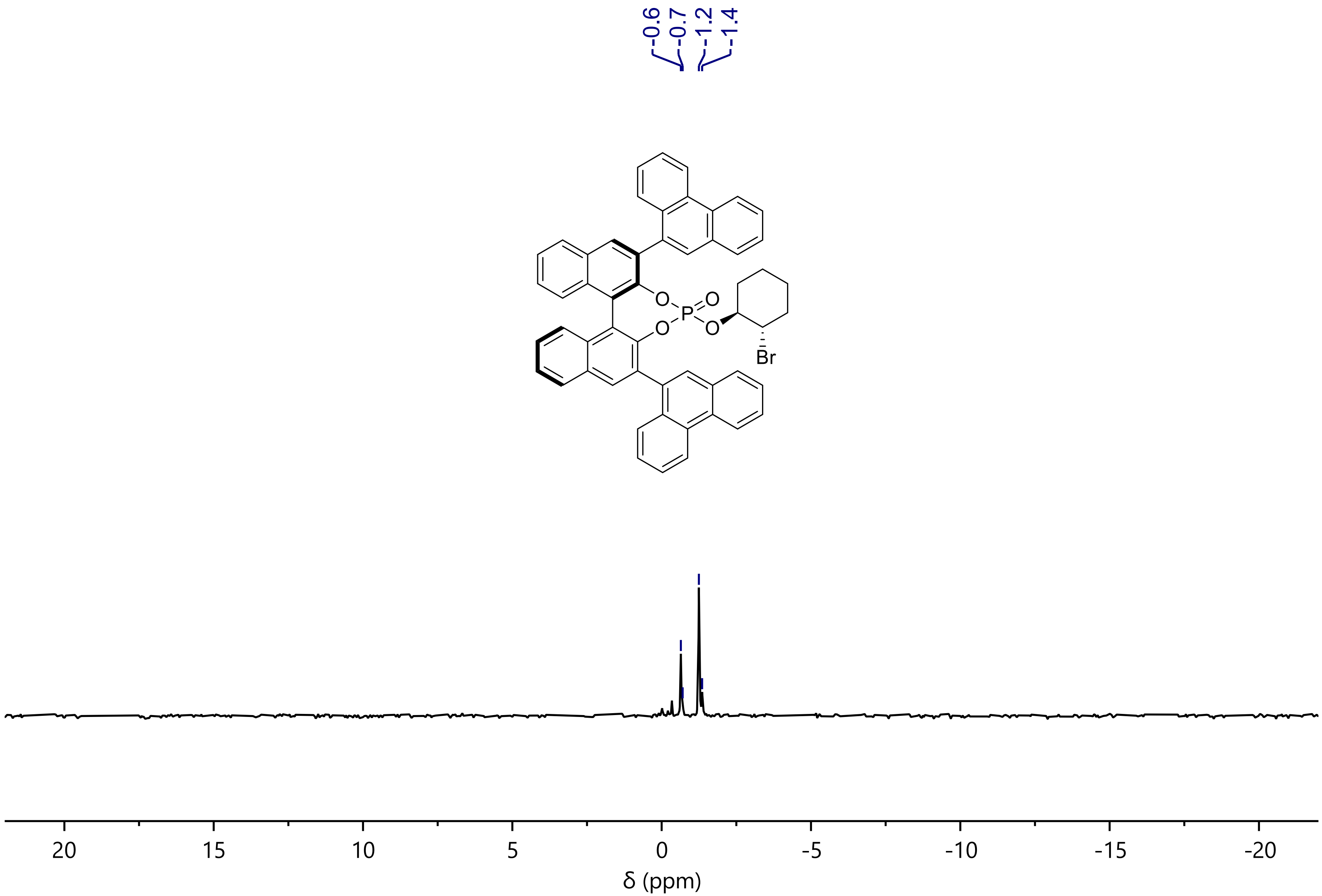

8. References

1. Spek, A. L. *PLATON* SQUEEZE: A Tool for the Calculation of the Disordered Solvent Contribution to the Calculated Structure Factors. *Acta Crystallogr. C* **2015**, *71*, 9–18. DOI: 10.1107/s2053229614024929.
